# Supplementary material for: Spaghetti to a Tree: A Robust Phylogeny for Terebelliformia (Annelida) Based on Transcriptomes, Molecular and Morphological Data
Source: Biology (Basel). 2020 Apr 6;9(4):73. doi: 10.3390/biology9040073 (PMC7236012; doi:10.3390/biology9040073)
Supplement: Supplementary file 1 [file biology-09-00073-s001.zip › Supplementary Tables.pdf]

Supplementary Tables

Supplementary Table S1: Voucher, sequencing and assembly statistics for the transcriptome dataset. Vouchers for all newly generated transcriptomes were deposited in the Scripps Institution of Oceanography Benthic Invertebrate Collection (SIO-BIC) and are highlighted in bold.

Species	Voucher	SRA Accession	Tech-nology	Read length	Read pairs raw	Read pairs after trimming	Assembled contigs	Avg contig length	Max contig length	N50	Number of loci
Alvinella caudata	---	SRR3665374	Hiseq 2000	PE-95	4,626,483	3,760,880	20,822	812	15,017	1024	3153
Alvinella pompejana	---	SRR3665375	Hiseq 2000	PE-95	16,861,102	6,072,375	27,023	902	17,332	1211	4133
***Amphicteis* cf. *gunneri* group 4**[2]	**A9430**	**SRR11434467**	Hiseq 4000	PE-100	47,931,393	45,147,588	79,401	1047	15,778	1514	6462
Amphisamytha carldarei	**A7710**	**SRR11434468**	Hiseq 4000	PE-100	39,266,357	33,542,842	63,988	1031	18,592	1545	6187
***Anobothrus* sp.**[3]	**A9432**	**SRR11434464**	Hiseq 4000	PE-100	38,399,649	35,811,389	71,665	1101	9,772	1641	6713
Eupolymnia crassicornis	**A9448**	**SRR11434465**	Hiseq 4000	PE-100	39,305,953	35,735,791	71,807	1512	16,618	2497	7534
Hypania invalida	---	SRR5590961	Hiseq 2500	PE-100	26,364,747	22,683,566	41,519	1281	25,342	2035	7099
Lanice conchilega	---	SRR5590962	Hiseq 2500	PE-100	7,153,766	6,973,497	44,774	948	17,627	1333	6427
Loimia bermudensis	**A9451**	**SRR11434463**	Hiseq 4000	PE-100	54,545,318	51,897,892	66,423	1228	13,748	1901	7073
Melinna oculata	**A9440**	**SRR11434462**	Hiseq 4000	PE-100	44,374,579	41,684,736	86,098	901	16,402	1247	5522
Nautalvinella irlandei[1]	---	SRR3665379	Hiseq 2000	PE-70	7,167,558	6,601,237	16,730	711	10,137	821	1913
Neoamphitrite robusta	**A9453**	**SRR11434461**	Hiseq 4000	PE-100	47,743,495	43,663,803	83,037	1390	17,418	2331	7505

Paralvinella fijiensis	---	SRR3665376	Hiseq 2500	PE-100	163,263,771	155,327,397	99,518	1315	29,173	2306	7268
Paralvinella grasslei	---	SRR3665377	Hiseq 2000	PE-95	29,031,987	20,798,332	43,837	1113	23,838	1696	5895
Paralvinella palmiformis	**A7728**	**SRR11434460**	Hiseq 4000	PE-100	40,277,701	34,514,297	58,263	1212	23,449	2003	6220
Pectinaria gouldii	---	SRR2057036	Hiseq 2000	PE	72,926,891	59,462,937	71,643	856	10,890	1099	3090
Polycirrus carolinensis	**A9444**	**SRR11434459**	Hiseq 4000	PE-100	29,062,749	26,915,272	62,519	1241	10,594	1976	6622
***Terebellides* sp.**[4]	**A9465**	**SRR11434458**	Hiseq 4000	PE-100	51,852,071	48,406,436	72,743	1174	16,512	1792	6972
Thelepus n.sp.	A5916	SRR2017640	HiSeq 2000	PE-100	41,581,952	37,783,007	86,315	456	16,862	505	3580
Trichobranchus roseus	**A9467**	**SRR11434466**	Hiseq 4000	PE-100	28,421,080	26,141,416	64,372	1107	10,169	1704	6064
Arenicola marina	---	SRR2005653	HiSeq 1000	PE-100	68,660,484	61,371,936	101,276	638	19,851	687	4109

[1] On NCBI SRA as *Paralvinella pandorae irlandei*. Our results indicate that it should be *Nautalvinella irlandei*.

[2] *Amphicteis gunneri* forms a cryptic species complex. This specimen falls in group 4 of an unpublished assessment (Jon Kongsrud, pers. comm.).

[3] This is an undescribed species informally called EP-B (Jon Kongsrud, pers. comm.).

[4] *Terebellides* is a cryptic species complex (Nygren et al. 2018) in the Atlantic. This specimen corresponds to species 25 in group C of Nygren et al. 2018.

Supplementary Table S2: Sequence and voucher information for Sanger-sequenced taxa. Sequences created for this study are highlighted in bold. Collection codes starting with A are from Scripps Institution of Oceanography Benthic Invertebrate Collection (SIO-BIC); collection codes starting with W are from the Australian Museum; CDF: Charles Darwin Foundation for the Galapagos Islands; ZMBN: Natural History Collections, University Museum of Bergen, Norway.

Taxon	Collection site	Voucher	COI	16S	18S	28S	H3
Alvinellidae							
Alvinella caudata Desbruyères & Laubier 1986	East Pacific Rise	A1092	JX423737	JX423669	JX423641	**MT166914**	**MT166723**
Alvinella caudata Desbruyères & Laubier 1986	East Pacific Rise	---	SRR3665374	SRR3665374	SRR3665374	SRR3665374	---
Alvinella pompejana Desbruyères & Laubier 1980	East Pacific Rise	A9429	AY645983	**MT166794**	**MT166855**	FP517935	**MT166724**
Alvinella pompejana Desbruyères & Laubier 1980	East Pacific Rise	---	SRR3665375	SRR3665375	SRR3665375	SRR3665375	---
Nautalvinella irlandei[1] (Desbruyères & Laubier 1986)	East Pacific Rise	---	SRR3665379	SRR3665379	SRR3665379	SRR3665379	---
Nautalvinella pandorae (Desbruyères & Laubier 1986)	Juan de Fuca Ridge	A1105	DQ270466	**MT166826**	**MT166884**	**MT166916**	**MT166764**
Paralvinella fijiensis Desbruyères & Laubier 1993	Lau Back-Arc Basin	ZMBN106600	MG270110	MG253099	MG253145	---	---
Paralvinella fijiensis Desbruyères & Laubier 1993	Lau Back-Arc Basin	---	SRR3665376	SRR3665376	SRR3665376	SRR3665376	SRR3665376
Paralvinella grasslei Desbruyères & Laubier 1982	East Pacific Rise	A1093A	**MT167009**	**MT166824**	AY577886	X80654	**MT166762**
Paralvinella grasslei Desbruyères & Laubier 1982	East Pacific Rise	---	SRR3665377	SRR3665377	SRR3665377	SRR3665377	SRR3665377
Paralvinella palmiformis Desbruyères & Laubier 1986	Juan de Fuca Ridge	A1104	**MT167010**	**MT166825**	AF168747	**MT166915**	**MT166763**
Paralvinella palmiformis Desbruyères & Laubier 1986	Juan de Fuca Ridge	A7728	SRR11434460	SRR11434460	SRR11434460	SRR11434460	SRR11434460
Paralvinella sulfincola Desbruyères & Laubier 1993 in Tunnicliffe et al. 1993	Juan de Fuca Ridge	---	FJ976042	FJ976042	JN936461	---	---
Ampharetidae							

Ampharetinae							
Amage auricula Malmgren 1866	Norwegian Sea	ZMBN 99281	---	MG253079	MG253131	---	---
Amage sculpta Ehlers 1908	Antarctica	---	---	MG253117	MG253162	---	---
Amagopsis klugei Pergament & Khlebovich 1964	Norwegian Sea	ZMBN 95455	MG270108	---	MG253173	---	---
Ampharete acutifrons (Grube 1860)	Iceland	---	HM473718	DQ779601	---	DQ779673	DQ779715
Ampharete falcata Eliason 1955	Norway, Skagerrak	ZMBN 95443	MG270099	MG253124	MG253169	---	---
Ampharete finmarchica (Sars 1864)	Svalbard, Spitsbergen	A1100	JX423738	JX423670	JX423642	**MT166917**	**MT166726**
Amphicteis dalmatica Hutchings & Rainer 1979	Australia, New South Wales	W199656	AF342678	---	---	AF342693	AF342700
Amphicteis gunneri (Sars 1835)	Norway, Glesvær	ZMBN 121368	MG270125	MG253105	MG253150	---	---
Amphicteis cf. *gunneri* group 2[2]	Norway, Trondheim fjord	A1107	**MT166980**	**MT166796**	**MT166856**	**MT166918**	**MT166727**
Amphicteis cf. *gunneri* group 4[3]	Sweden, Koster area	A9430	SRR11434467	SRR11434467	SRR11434467	SRR11434467	SRR11434467
Amphicteis obscurior Chamberlin 1919	Mexico, Gulf of California	A9431	**MT166981**	**MT166797**	---	**MT166919**	**MT166728**
Amphicteis ninonae Jirkov 1985	Svalbard, Spetsbergen	ZMBN 95441	KX497038	KX513561	---	---	---
Amphisamytha carldarei Stiller et al. 2013	Juan de Fuca Ridge	A7710	SRR11434468	SRR11434468	SRR11434468	SRR11434468	SRR11434468
Amphisamytha jacksoni Stiller et al. 2013[4]	Galapagos Ridge	CDF NA067-045-05	**MT166982**	**MT166798**	**MT166857**	**MT166920**	**MT166729**
Amythas membranifera Benham 1921	Scotia Arc	A2947	**MT166983**	**MT166799**	**MT166858**	**MT166921**	**MT166730**
Anobothrus gracilis (Malmgren 1866)	Norway, Trondheim fjord	A1106	JX423739	JX423671	JX423643	AF501670	**MT166731**
Anobothrus laubieri (Desbruyères 1978)	---	ZMBN 89833	---	MG253080	MG253132	---	---
Anobothrus sp. EP-B[5]	USA, California, Whale "Ruby"	A9432	SRR11434464	SRR11434464	SRR11434464	SRR11434464	SRR11434464
Asabellides californica Hilbig 2000	---	A9433	**MT166986**	**MT166802**	**MT166860**	**MT166922**	**MT166734**

Auchenoplax crinita Ehlers 1887	USA, New England	---	FJ976041	FJ976041	DQ790077	DQ790026	---
Auchenoplax worsfoldi Jirkov & Leontovich 2013	Mauritania	ZMBN 95430	MG270100	MG253118	MG253165	---	---
Eclysippe eliasoni (Day 1973)	Norway, Trondheim fjord	A1108	JX423766	JX423678	JX423648	**MT166924**	**MT166737**
Eclysippe vanelli (Fauvel 1936)	Mauritania	ZMBN 95433	MG270103	MG253119	MG253166	---	---
Glyphanostomum pallescens (Théel 1879)	Iceland	---	MG270091	MG253092	MG253142	---	---
Grassleia cf. *hydrothermalis* Solís-Weiss 1993	Mexico, Gulf of California	A6137	KX497032	KX513552	KX13568	---	---
Hypania invalida (Grube 1860)	Germany, Magdeburg	---	SRR5590961	SRR5590961	**---**	SRR5590961	SRR5590961
Lysippe labiata Malmgren 1866	Svalbard, Billefjord	A9434 / ZMBN 95444	**MT167003**	MG253125	**MT166876**	**MT166925**	**MT166752**
Mugga wahrbergi Eliason 1955	Sweden, Gullmarsfjorden	A1118	JX423768	JX423680	JX423650	**MT166926**	**MT166757**
Neosabellides elongatus (Ehlers 1912)	Scotia Arc	A9435	**MT167007**	**MT166821**	**MT166881**	**MT166927**	**MT166759**
Neosabellides lizae Alvestad & Budaeva 2015	Australia, Lizard Island	W45180	MG270089	---	MG253134	---	---
Noanelia sp. A	Icelandic Basin	---	MG270121	MG253098	MG253144	---	---
Paralysippe annectens (Moore 1923)	USA, California, "Fossil Hill"	A9436	**MT167011**	**MT166827**	**MT166885**	**MT166928**	**MT166765**
Paramytha ossicola Queirós et al. 2016	Portugal, Setùbal Canyon	ZMBN 107234	---	KX513549	KX513565	---	---
Paramytha schanderi Kongsrud et al. 2016	Mid-Atlantic Ridge, Loki's Castle	ZMBN 87820	KX497035	KX513555	KX513571	---	---
Pavelius smileyi Kongsrud et al. 2016	Mid-Atlantic Ridge, Loki's Castle	ZMBN 87807	KX497034	KX513554	KX513570	---	---
Pavelius uschakovi Kuznetsov & Levenstein 1988	Russia, Okhotsk Sea	ZMBN 108241	KX497033	KX513553	KX513569	---	---
Pavelius sp. EP-B[6]	USA, Oregon, Hydrate Ridge	A1980 / A2065	**MT167012**	**MT166828**	**MT166886**	**MT166923**	**MT166766**
Sabellides octocirrata (Sars 1835)	Norway, Trondheim fjord	A1109	JX423770	JX423682	JX423652	**MT166929**	**MT166778**

Samytha sexcirrata (Sars 1856)	Norway, Trondheim fjord	A1110	**MT167021**	**MT166839**	**MT166897**	**MT166930**	**MT166779**
Samythella neglecta Wollebaek 1912	Norwegian Sea	ZMBN 99276	MG270113	KX513551	KX513567	---	---
Sosane sulcata Malmgren 1866	Sweden, Vattenholmen	A1120	MG270078	**MT166841**	**MT166899**	**MT166931**	**MT166781**
Sosane wireni (Hessle 1917)	Norway, Bergen area	ZMBN 95447	KX497039	KX513562	KX513577	---	---
Melinnidae							
Isolda bipinnata Fauchald 1977	Belize	A9437	**MT166993**	**MT166809**	**MT166867**	**MT166932**	**MT166742**
Isolda pulchella Müller in Grube 1858	Morocco	ZMBN 105697	MG270120	MG253082	MG253136	---	---
Melinna albicincta Mackie & Pleijel 1995	Norway, Trondheim Fjord	A1113	JX423767	JX423679	JX423649	**MT166933**	**MT166753**
Melinna cristata (Sars 1851)	Sweden, Gullmaren	A9438	GQ229117	DQ779624	**MT166877**	**MT166934**	**MT166754**
Melinna heterodonta (Moore 1923)	USA, California	A4546	**MT167004**	**MT166818**	**MT166878**	**MT166935**	**MT166755**
Melinna oculata Hartman 1969	USA, California	A9439	**MT167005**	**MT166819**	**MT166879**	**MT166936**	**MT166756**
Melinna oculata Hartman 1969	USA, California	A9440	SRR11434462	SRR11434462	SRR11434462	SRR11434462	---
Pectinariidae							
Pectinaria auricoma (Müller 1776)	Sweden, Kristineberg	A1004	**MT167013**	DQ319857	**MT166887**	AF501668	**MT166767**
Pectinaria gouldii (Verrill 1874)	---	---	FJ976040	FJ976040	DQ790091	DQ790054	---
Pectinaria gouldii (Verrill 1874)	Massachusetts, USA	---	SRR2057036	SRR2057036	SRR2057036	SRR2057036	---
Pectinaria granulata (Linné 1767)	Iceland	A9441	HQ970907	**MT166829**	AY577890	DQ209239	**MT166768**
Pectinaria hyperborea (Malmgren, 1866)	Russia, Kandalaksha Bay	WS0244/ WS668	GU672461	---	---	---	KP113607
Pectinaria koreni (Malmgren 1866)	English Channel / North Sea	---	DQ319528	DQ319865	JN936463	---	---
Petta pusilla Malmgren 1866	Sweden, Kostergrund	A1122	**MT167014**	**MT166830**	**MT166888**	**MT166937**	**MT166769**
Terebellidae							
Terebellinae							

Amaeana trilobata (Sars 1863)	Norway, Trondheimsfjord	A9442	**MT166979**	**MT166795**	---	**MT166938**	**MT166725**
Artacama crassa Hartman 1967	Scotia Arc	A3151	**MT166984**	**MT166800**	**MT166859**	**MT166947**	**MT166732**
Artacama proboscidea Malmgren 1866	Sweden, Kostergrund	A1125	**MT166985**	**MT166801**	AY344666	**MT166948**	**MT166733**
Axionice maculata (Dalyell 1853)	Svalbard, Hinlopenstretet	A1097	**MT166987**	**MT166803**	**MT166861**	**MT166949**	**MT166735**
Biremis cf. *blandi* Polloni et al. 1973	Mexico, Alarcon Rise	A7707	**MT166988**	**MT166804**	**MT166862**	**MT166939**	**MT166736**
Eupolymnia crassicornis (Schmarda 1861)	Belize	A9446	**MT166989**	**MT166805**	**MT166863**	**MT166950**	**MT166738**
Eupolymnia crassicornis (Schmarda 1861)	Belize	A9448	SRR11434465	SRR11434465	SRR11434465	SRR11434465	SRR11434465
Eupolymnia nebulosa (Montagu 1818)	France, Banyuls	A1080	**MT166990**	**MT166806**	**MT166864**	**MT166951**	**MT166739**
Eupolymnia rullieri Londoño-Mesa, 2009	Belize	A9447	**MT166991**	**MT166807**	**MT166865**	**MT166952**	**MT166740**
Hauchiella renilla Hutchings & Glasby 1986	Australia, South Australia	A1116	**MT166992**	**MT166808**	**MT166866**	**MT166940**	**MT166741**
Lanassa venusta (Malm 1874)	Svalbard, Raudfjord	A1099	**MT166994**	**MT166810**	**MT166868**	**MT166953**	**MT166743**
Lanice conchilega (Pallas 1766)	England, Scilly Islands	A1115	**MT166995**	AY340466	X79873	AY340403	**MT166744**
Lanice conchilega (Pallas 1766)	France, Roscoff	---	SRR5590962	SRR5590962	SRR5590962	SRR5590962	---
Lanicola lobata Hartmann-Schröder 1986	Australia, Tasmania	A9450	**MT166997**	**MT166812**	**MT166870**	**MT166955**	**MT166746**
Laphania boecki Malmgren 1866	Svalbard, Billefjord	A1095	**MT166998**	**MT166813**	**MT166871**	**MT166956**	**MT166747**
Leaena ebranchiata (Sars 1865)	Svalbard, Billefjord	A1094	**MT166999**	**MT166814**	**MT166872**	**MT166957**	**MT166748**
Loimia bermudensis Verrill 1900	Belize	A9451	**MT167000**	**MT166815**	**MT166873**	**MT166958**	**MT166749**
Loimia bermudensis Verrill 1900	Belize	A9451	SRR11434463	SRR11434463	SRR11434463	SRR11434463	SRR11434463
Loimia salazari Londoño-Mesa & Carrera-Parra 2005	---	---	AY040704	---	AY040690	---	---
Loimia minuta Treadwell 1929	Belize	A9452	**MT167001**	**MT166816**	**MT166874**	**MT166959**	**MT166750**

Lysilla cf. *pacifica* Hessle 1917	New Caledonia, Lifou Island	A1114	**MT167002**	**MT166817**	**MT166875**	**MT166941**	**MT166751**
Neoamphitrite affinis (Malmgren 1866)	Sweden, Gullmarsfjord	A1117	**MT167006**	**MT166820**	**MT166880**	**MT166960**	**MT166758**
Neoamphitrite robusta (Johnson 1901)	USA, California	A9453	SRR11434461	SRR11434461	SRR11434461	SRR11434461	SRR11434461
Paraeupolymnia carus Young & Kritzler 1987	Belize	A9449	**MT166996**	**MT166811**	**MT166869**	**MT166954**	**MT166745**
Pista australis Hutchings & Glasby 1988	Australia, New South Wales	W24390	AF342682	---	---	AF185159	AF185242
Pista cristata (Müller 1776)	Norway, Trondhejmsfjord	ZMBN 116032	MG270116	MG253107	MG253153	---	---
Polycirrus arcticus Sars 1865	Svalbard, Billefjord	A1096	**MT167015**	**MT166831**	**MT166889**	**MT166942**	**MT166770**
Polycirrus californicus Moore 1909	USA, California	A9443	**MT167016**	**MT166832**	**MT166890**	**MT166943**	**MT166771**
Polycirrus carolinensis Day 1973	Belize	A1101	---	**MT166833**	**MT166891**	**MT166944**	**MT166772**
Polycirrus carolinensis Day 1973	Belize	A9444	SRR11434459	SRR11434459	SRR11434459	SRR11434459	SRR11434459
Polycirrus eximius (Leidy 1855)	USA, North Carolina	A9445	**MT167017**	**MT166834**	**MT166892**	**MT166945**	**MT166773**
Polycirrus norvegicus Wollebæk 1912	Norway, Trondheim fjord	A1111	---	**MT166835**	**MT166893**	**MT166946**	**MT166774**
Proclea malmgreni (Ssolowiew 1899)	Sweden, Singlefjord	A1121	**MT167018**	**MT166836**	**MT166894**	**MT166961**	**MT166775**
Reteterebella cf. *aloba* Hutchings & Glasby 1988	---	A9454	**MT167019**	**MT166837**	**MT166895**	**MT166962**	**MT166776**
Scionella lornensis Pearson 1969	Sweden, Gullmarsfjord	A1119	**MT167022**	**MT166840**	**MT166898**	**MT166963**	**MT166780**
Spinosphaera hutchingsae Londoño-Mesa 2004	Belize	A9455	**MT167023**	**MT166842**	**MT166900**	**MT166964**	**MT166782**
Terebella lapidaria Linnaeus 1767	England, English Channel	A1102	JX423771	JX423683	JX423653	**MT166965**	**MT166785**
Thelepodinae							
Rhinothelepus lobatus Hutchings 1974	Australia, New South Wales	W49044	**MT167020**	**MT166838**	**MT166896**	**MT166966**	**MT166777**
Streblosoma kaia Reuscher et	North Fiji Basin,	A9456	**MT167024**	**MT166843**	**MT166901**	**MT166967**	**MT166783**

al. 2011	White Lady						
Streblosoma pacifica Hilbig 2001	USA, California	A9457	**MT167025**	**MT166844**	**MT166902**	**MT166968**	**MT166784**
Thelepus antarcticus Kinberg 1866	Scotia Arc	A2944	**MT167028**	**MT166847**	**MT166905**	**MT166969**	**MT166788**
Thelepus australiensis Hutchings & Smith 1997	Australia, Whitsunday Islands	A9458	**MT167029**	**MT166848**	**MT166906**	**MT166970**	**MT166789**
Thelepus cincinnatus (Fabricius 1780)	Norway, Trondheim fjord	A9460/ SMNH 81625	**MT167031**	DQ779636	AY611462	DQ779712	DQ779769
Thelepus cf. *pascua* (Fauchald 1977)	Indonesia, Raja Ampat	A9459	**MT167030**	**MT166849**	**MT166907**	**MT166971**	**MT166790**
Thelepus n.sp.	USA, California	A5916	SRR2017640	SRR2017640	SRR2017640	SRR2017640	SRR2017640
Thelepus n.sp.2	USA, California	A9461	**MT167032**	**MT166850**	**MT166908**	**MT166972**	**MT166791**
Thelepus verrilli (Treadwell 1911)	Belize	A9462	**MT167033**	**MT166851**	**MT166909**	---	**MT166792**
Trichobranchidae							
Octobranchus lingulatus (Grube 1863)	France, Banyuls	A1103	**MT167008**	**MT166822**	**MT166882**	**MT166974**	**MT166760**
Octobranchus sp.[7]	USA, California, Whale Pedro	A9463	**---**	**MT166823**	**MT166883**	**MT166973**	**MT166761**
Terebellides atlantis Williams 1984	South of Iceland	SMF 24678	MG025259	MG025491	---	MG025418	---
Terebellides bigeniculatus Parapar, Moreira & Helgason 2011	South of Iceland	SMF 24636	MG025318	MG025500	---	MG025427	---
Terebellides gracilis Malm 1874	Norway	NTU-V M-68196	MG024998	MG025452	---	MG025368	---
Terebellides irinae Gagaev 2009	Arctic Ocean	ZMBN 116500	MG025342	MG025506	---	MG025432	---
Terebellides shetlandica Parapar, Moreira & O'Reilly 2016	Sweden, Kristineberg	A1143	**MT167026**	**MT166845**	**MT166903**	**MT166976**	**MT166786**
Terebellides sp. LA1[8]	USA, California	A9464	**MT167027**	**MT166846**	**MT166904**	**MT166975**	**MT166787**
Terebellides sp.[9]	USA, Washington	A9465	SRR11434458	SRR11434458	SRR11434458	SRR11434458	SRR11434458
Terebellides stroemii Sars 1835	Skagerrak, North Sea	ZMBN 116344	MG025097	MG025462	---	MG025380	---
Terebellides williamsae Jirkov 1989	Barents Sea	ZMBN 116258	MG024977	MG025448	---	MG025363	---

Trichobranchus cf. *bibranchiatus* Moore 1903	Indonesia, Raja Ampat	A7289	**MT167034**	**MT166852**	**MT166910**	**MT166977**	**MT166793**
Trichobranchus hancocki (Hartman 1955)	USA, California	A9466	**MT167035**	**MT166853**	**MT166911**	---	---
Trichobranchus roseus (Malm 1874)	Sweden, Gasoriken	A1084	**MT167036**	**MT166854**	**MT166912**	**MT166978**	---
Trichobranchus roseus (Malm 1874)	Sweden, Koster area	A9467	SRR11434466	SRR11434466	SRR11434466	SRR11434466	SRR11434466
Trichobranchus tribranchiata (Hutchings & Peart 2000)	Australia, South Australia	A1123	**MT167037**	DQ779605	**MT166913**	DQ779677	DQ779720
Outgroup							
Arenicola marina (Linnaeus 1758)	---	---	HQ691225	HQ691210	HQ691215	HQ691220	DQ779718

[1] On NCBI SRA as *Paralvinella pandorae irlandei.* Our results indicate that it should be *Nautalvinella irlandei*.

[2] *Amphicteis gunneri* forms a cryptic species complex. This specimen falls in group 2 of an unpublished assessment (Jon Kongsrud, pers. comm.).

[3] *Amphicteis gunneri* forms a cryptic species complex. This specimen falls in group 4 of an unpublished assessment (Jon Kongsrud, pers. comm.).

[4] We are thankful to the Ocean Exploration Trust as well as the pilots and crew aboard the E/V Nautilus during cruise NA064 for their assistance in sample collection and exploration using the Hercules ROV. Further acknowledgements and thanks go out to the Charles Darwin Foundation and the Galapagos National Park Directorate for their collaboration and assistance in the exploration of the Galapagos Platform conducted under research permit No. PC-45-15. We also gratefully recognize the Government of Ecuador via the Ecuadorian Navy for permission to operate in their territorial waters.

[5] This is an undescribed species informally called *Anobothrus* sp. EP-B (Jon Kongsrud, pers. comm.).

[6] This is an undescribed species informally called *Pavelius* sp. EP-B (Jon Kongsrud, pers. comm.).

[7] This is an undescribed species that has been informally referred to as "*Bizzarobranchus*" or "*genus B*" (SCAMIT, https://preview.tinyurl.com/wnmmnv5).

[8] This is an undescribed species that has been informally referred to as *Terebellides* "sp. LA1" (SCAMIT Newsletter Vol 25(5), https://preview.tinyurl.com/tqayhud).

[9] *Terebellides* is a cryptic species complex (Nygren et al. 2018) in the Atlantic. This specimen corresponds to species 25 in group C of Nygren et al. 2018.
