# Supplementary material for: Spaghetti to a Tree: A Robust Phylogeny for Terebelliformia (Annelida) Based on Transcriptomes, Molecular and Morphological Data
Source: Biology (Basel). 2020 Apr 6;9(4):73. doi: 10.3390/biology9040073 (PMC7236012; doi:10.3390/biology9040073)
Supplement: Supplementary file 1 [file biology-09-00073-s001.zip › Supplementary file 1.pdf]

Supplementary file 1: Commands used for phylogenetic reconstruction of transcriptome trees, molecular partition, morphological partition and the total evidence matrix. Gene tree heterogeneity and subsetting criteria.

All used and resulting files can be found on the data repository (DOI 10.17632/7vrfjhbdxs.1).

Supplementary acknowledgements

For the specimen of *Amphisamytha jacksoni* from the Galapagos Ridge:
We are thankful to the Ocean Exploration Trust as well as the pilots and crew aboard the E/V Nautilus during cruise NA064 for their assistance in sample collection and exploration using the Hercules ROV. Further acknowledgements and thanks go out to the Charles Darwin Foundation and the Galapagos National Park Directorate for their collaboration and assistance in the exploration of the Galapagos Platform conducted under research permit No. PC-45-15. We also gratefully recognize the Government of Ecuador via the Ecuadorian Navy for permission to operate in their territorial waters.

Commands used for phylogenetic inference and supplementary analyses

Table of contents

- Commands used for phylogenetic inference and supplementary analyses
  - Transcriptome dataset
    - ML analyses on concatenated matrices with IQ-TREE
    - Coalescent based species tree with ASTRAL
  - Gene tree heterogeneity
  - Identification of potentially extraneous sequences
  - Sanger sequenced genes and morphology
    - Molecular five gene dataset
    - Morphological matrix
    - Molecular five gene matrix and morphology, unconstrained
    - Molecular five gene matrix and morphology, constrained to transcriptome topology
    - Molecular five gene matrix, constrained to transcriptome topology

Transcriptome dataset

The data repository (DOI 10.17632/7vrfjhbdxs.1) contains

- Amino acid alignment files (fasta) for all 13,215 loci
- Amino acid alignment files (fasta) for all filtered 12,674 loci, which were used for downstream phylogenetic analyses
- Maximum likelihood (ML) gene trees for all 12,674 loci including IQ-TREE log file with the chosen model and parameters
- All 12,674 gene trees in one file used for ASTRAL species tree estimation
- ASTRAL species tree from summarizing 12,674 loci
- Concatenated matrices, corresponding species trees and IQ-TREE log files for 80%, 90%, 95% and 100% matrices.
- Heterogeneity assessments, see below
- Identification of potentially extraneous sequences, see below

We also show the resulting tree files here for easier comparison.

ML analyses on concatenated matrices with IQ-TREE

Transcriptome dataset, 80% occupancy, run on CIPRES (infile.txt: 80p.fa)

```
iqtree -s infile.txt -bb 1000 -bnni -st AA -m LG+G4 -pre 80p -nt AUTO
```

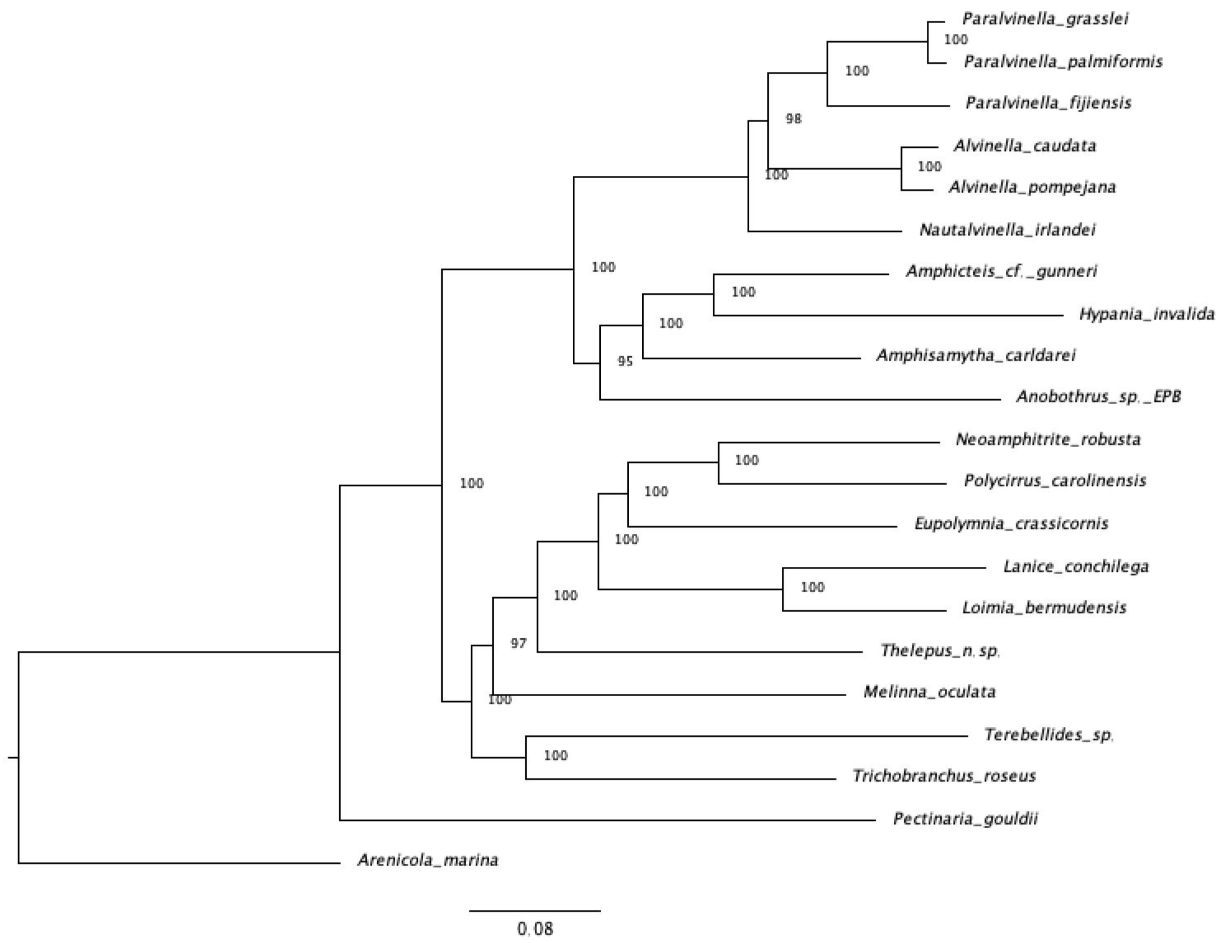

Supplementary Figure S1: ML tree from the 80% matrix of loci at least 17 taxa. This is the topology shown in main text Figure 1.

Transcriptome dataset, 90% occupancy, run on CIPRES (infile.txt: 90p.fa)

```
iqtree -s infile.txt -bb 1000 -bnni -st AA -m LG+G4 -pre 90p -nt AUTO
```

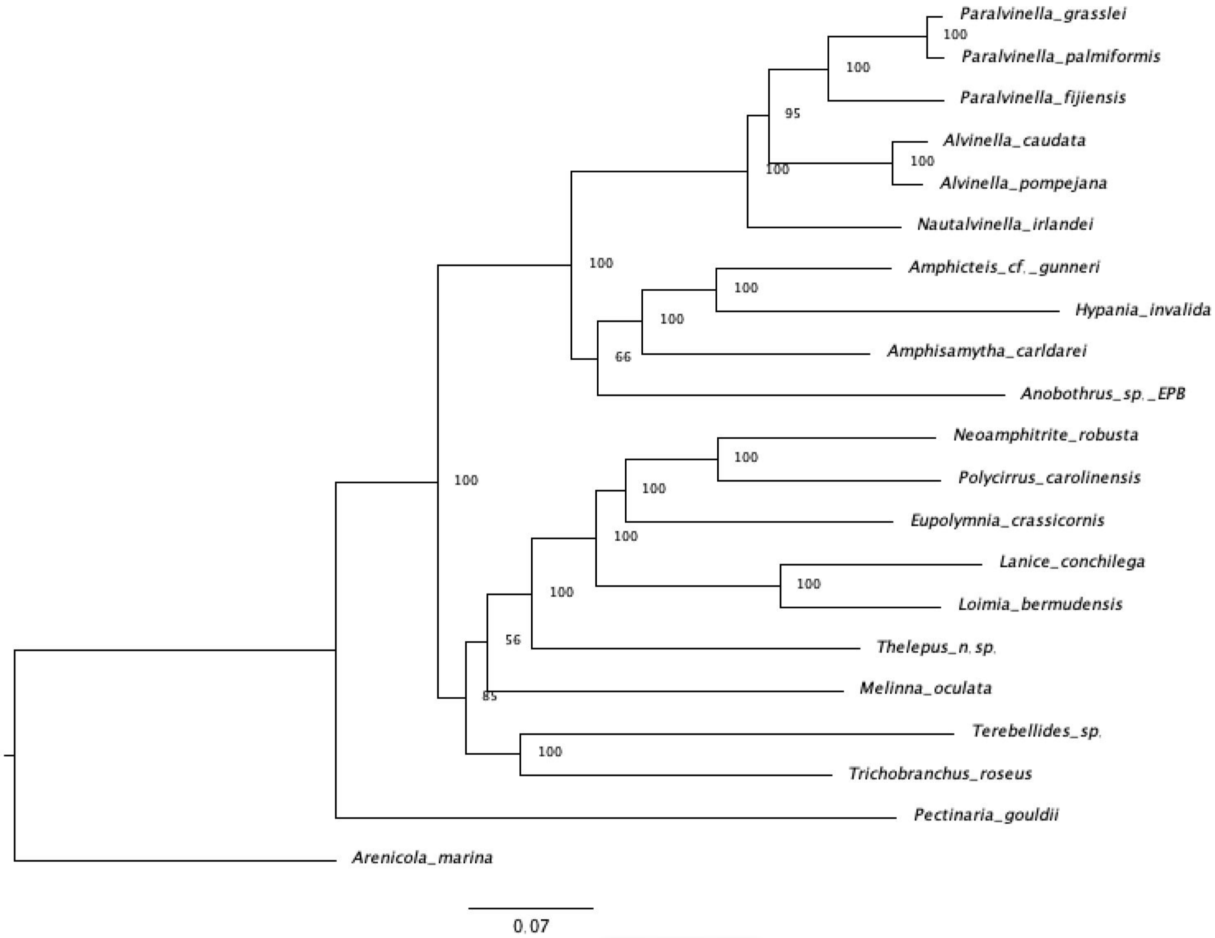

Supplementary Figure S2: ML tree from the 90% matrix with at least 19 taxa. The topology is identical to the one in main text Figure 1 and the bootstrap supports are indicated in Figure 2.

Transcriptome dataset, 95% occupancy, run on CIPRES (infile.txt: 95p.fa)

```
iqtree -s infile.txt -bb 1000 -bnni -st AA -m LG+G4 -pre 95p -nt AUTO
```

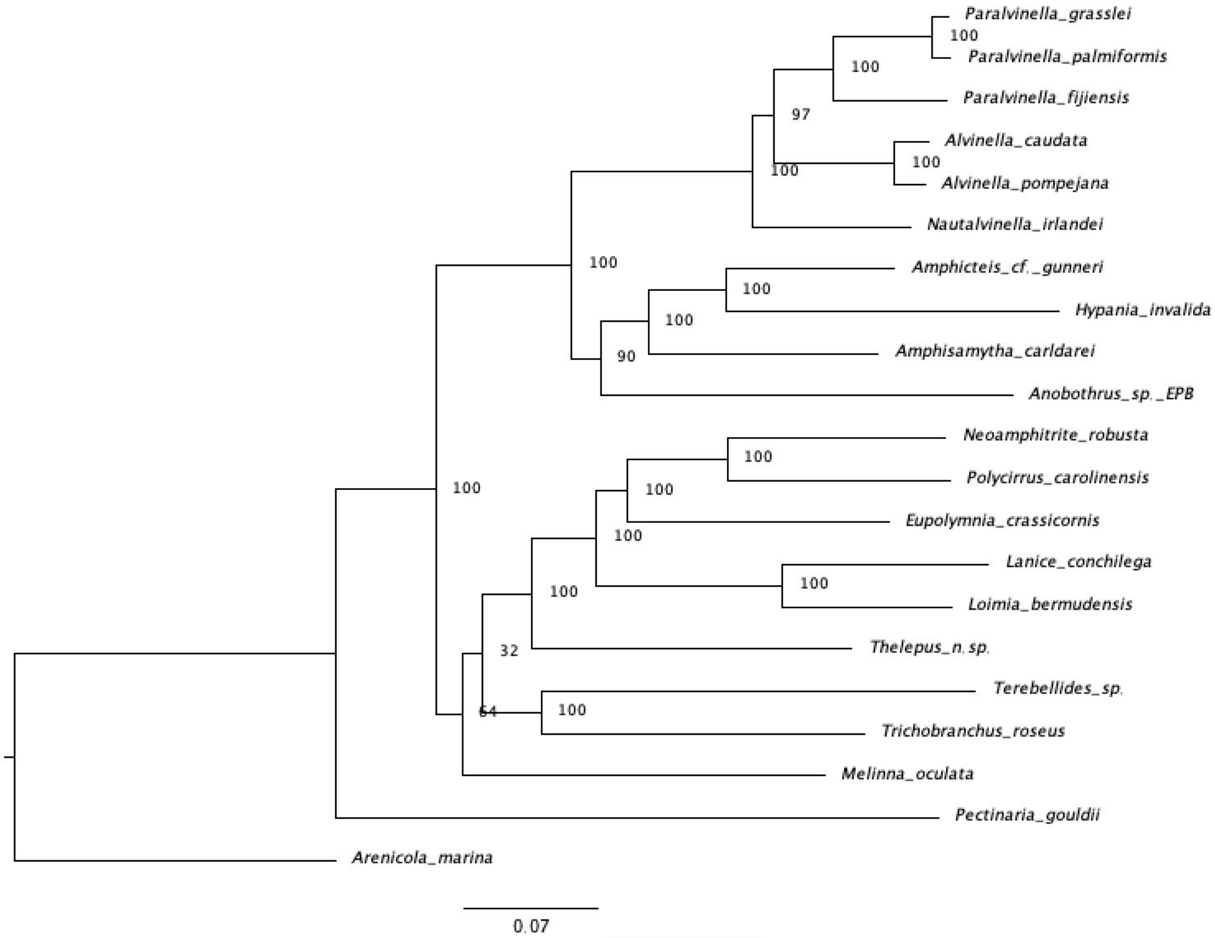

Supplementary Figure S3: ML tree from the 95% matrix with at least 20 taxa.

Transcriptome dataset, 100% occupancy, run on CIPRES (infile.txt: 100p.fa)

```
iqtree -s infile.txt -bb 1000 -bnni -st AA -m LG+G4 -pre 100p -nt
AUTO
```

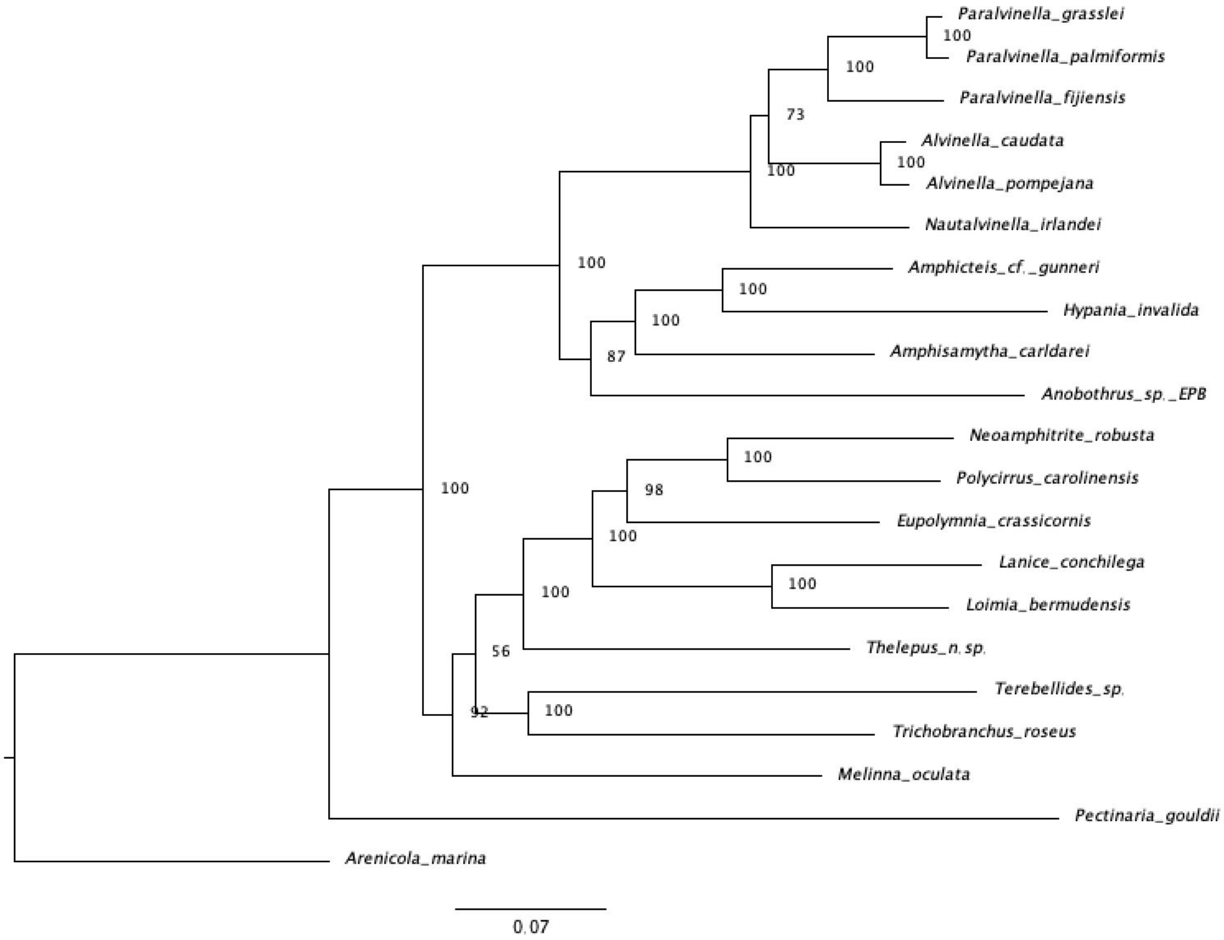

Supplementary Figure S4: ML tree from the complete 100% matrix with all taxa.

Individual gene trees for 10,746 alignments

Run ModelFinder on each alignment ($INPUT.fa), then ML tree search with IQ-TREE

```
iqtree -s $INPUT.fa -m MFP -bb 1000 -nt AUTO
```

Coalescent based species tree with ASTRAL

```
java -jar astral.5.14.3.jar -i
all_12674_filtered_genes_iqtree.gene.trees -o
all_12674_filtered_genes_iqtree.species.tre
```

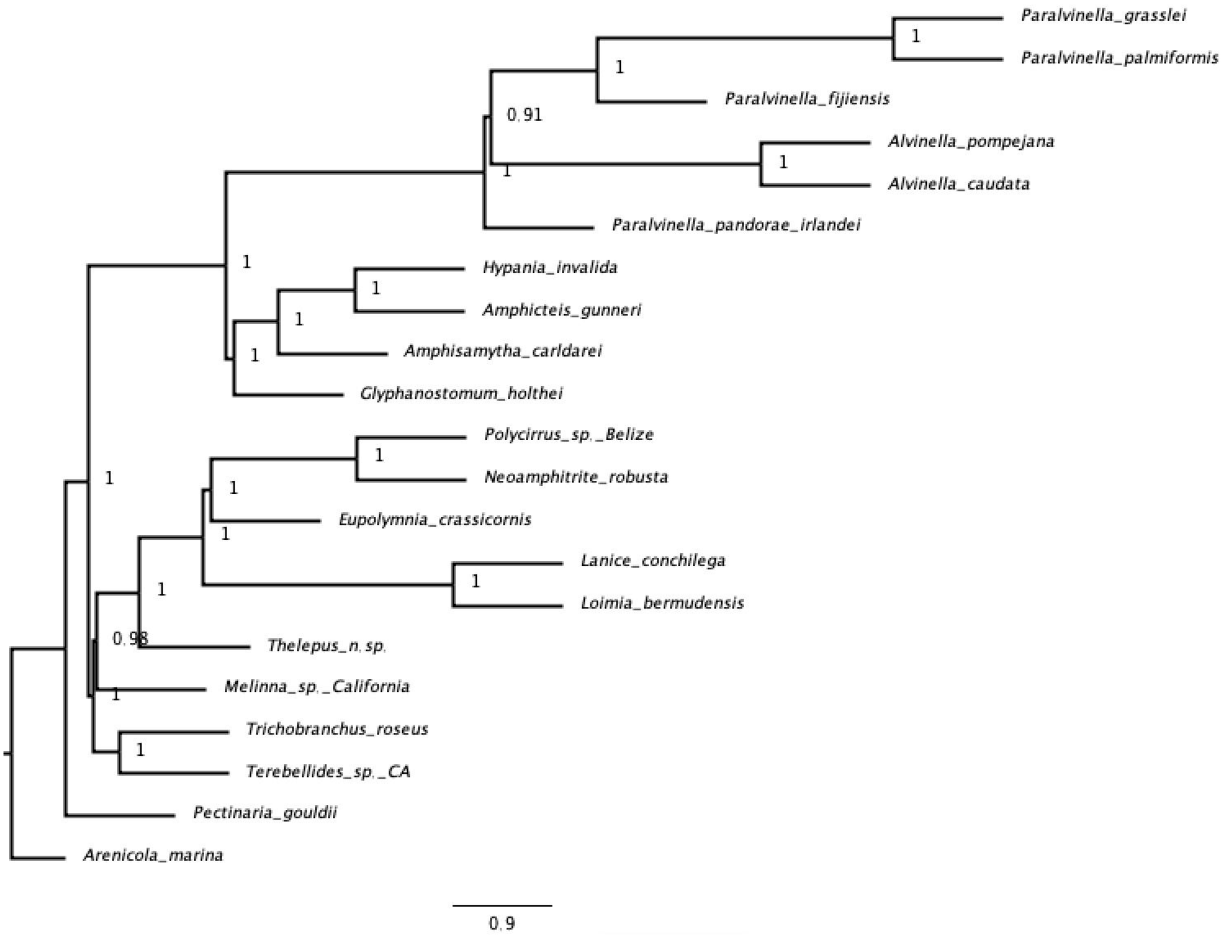

Supplementary Figure S5: Coalescent-based species tree from ASTRAL based on 12,674 gene trees. Node labels are local posterior probabilities (PP). The topology is identical to the one in main text Figure 1 and the PP values are shown in the figure.

Gene tree heterogeneity

The data repository (DOI 10.17632/7vrfjhbdxs.1) contains

- Statistics for i) long branch scores, ii) average bootstrap support and iii) heterogeneity test (Symtest in IQ-TREE v. 2) for each locus (including partition file with models that was used in the test)
- List of loci that passed each of the criteria individually, and all criteria together
- A file of all gene trees of loci that passed each of the criteria individually, and all criteria together
- A file of the resulting ASTRAL species trees summarizing all gene trees of loci that passed each of the criteria individually, and all criteria together

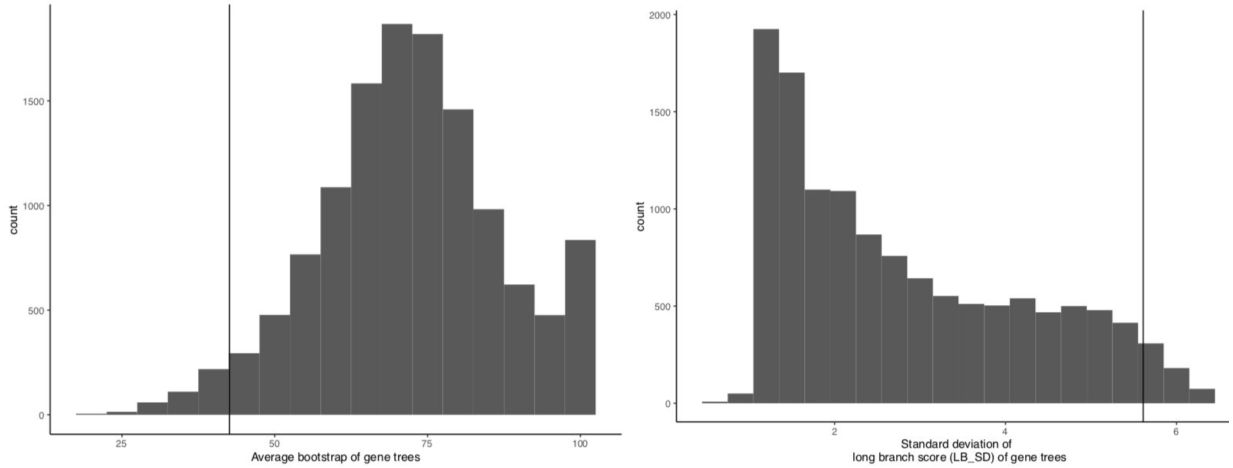

Supplementary Figure S6: Distribution of average bootstrap support and branch lengths. Left: Histogram of average bootstrap support. Right: Histogram of standard deviation of the long branch scores. The black vertical lines indicate extreme values 2 standard deviations from the mean. These gene trees were excluded to test their influence on species tree reconstruction.

Supplementary Table S3: Summary of metrics used to filter gene trees and resulting local posterior probability (PP) in key nodes. Model violations were tested with IQ-TREE v. 2 and loci were excluded if they had a P-value<0.05 in the maximum test of symmetry (SymPval). For the branch length heterogeneity (i.e. standard deviation in branch lengths for each gene tree) and average bootstrap, gene trees were excluded if their values were 2 standard deviations from the mean. The resulting species trees from ASTRAL (available from the repository) were topologically identical to the main hypothesis in Figure 1. They only differed in PP support of two clades, i) (*Alvinella_pompejana, Alvinella_caudata, Paralvinella_grasslei, Paralvinella_palmiformis, Paralvinella_fijiensis*), and ii) (*Melinna_oculata*, Terebellidae), for which values are given in the last two columns.

Metric	Cutoff for exclusion	Number of gene trees excluded	Number of gene trees remaining	PP *Alvinella*+*Paralvinella*	PP *Melinna*+Terebellidae
Adherence to substitution model assumptions	SymPval<0.05	77	12,597	0.91	0.98
Low heterogeneity in branch lengths	Long branch SD>5.61	562	12,112	0.84	0.99
High bootstrap support	Average bootstrap<42.6	407	12,267	0.92	0.98
Remove all extremes	All above	1040	11,634	0.81	0.99

Identification of potentially extraneous sequences

1) We removed identical sequences, which can be signs of potential cross contamination. Table alignments_with_identical_sequences.txt on the repository (DOI 10.17632/7vrfjhbdxs.1) gives the identical sequence pairs for each of the 456 affected alignments. Note that identical sequences are also more likely between closely related species, which may explain the high proportion of identical sequences between Alvinellidae.

The corresponding branches ($taxa_to_remove) were removed from the gene trees ($treefile):

```
pxrmt -t $treefile -n $taxa_to_remove >> removed_identical.gene.trees
```

Following the pruning, 49 gene trees had less than 4 taxa, and were removed (such trees cannot be analyzed by ASTRAL).

2) We ran TreeShrink using default settings

```
python TreeShrink/run_treeshrink.py -t removed_identical.gene.trees
-o output_TreeShrink
```

Outputs cleaned gene tree file to be analyzed with ASTRAL

```
java -jar astral.5.14.3.jar -i
output_TreeShrink/removed_identical.gene_0.05.trees -o
removed_identical.treeshrink_0.05.species.tre
```

The resulting species tree was identical to the topology in Figure 1 with better posterior probability support on the node (*Alvinella_pompejana, Alvinella_caudata, Paralvinella_grasslei, Paralvinella_palmiformis, Paralvinella_fijiensis*). It is possible that the higher support on this node was caused by the removal of many identical sequences in Alvinellidae, hence reducing the number of polytomies in the tree. As mentioned above these identical sequences may be more likely a sign of the relatively close evolutionary relationships among Alvinellidae than of cross-contamination. This is supported by the fact that *Paralvinella palmiformis* could not have cross-contaminated any of the other Alvinellidae because they were sequenced as part of different studies; nonetheless the sample had many identical sequences to other alvinellids, mostly to its closest relative *P. grasslei*.

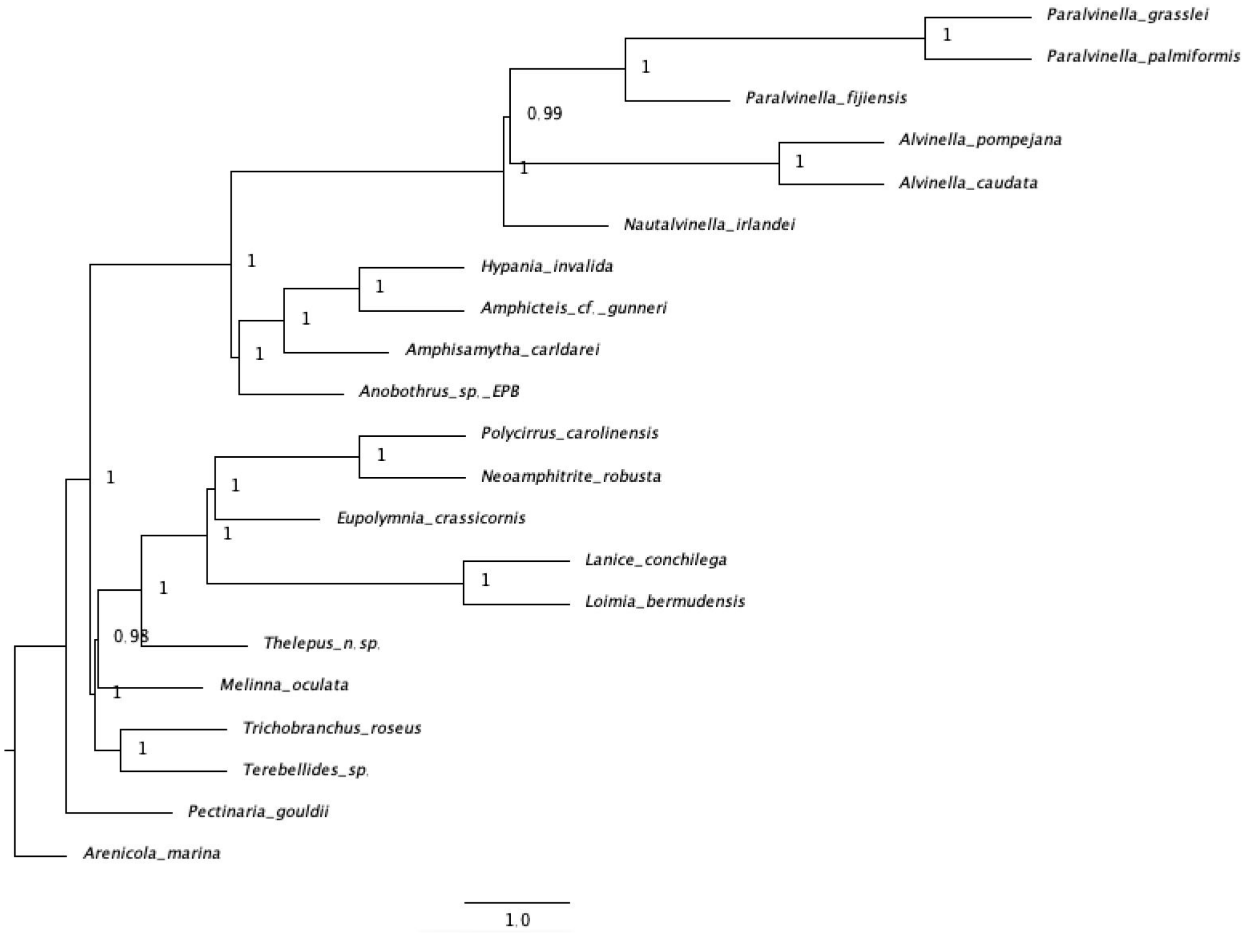

Supplementary Figure S7: Coalescent-based species tree from ASTRAL based on gene trees after cleaning for i) identical sequences and ii) for unusually long branches. Node labels are local posterior probabilities. The topology is identical to the one in main text Figure 1.

Sanger sequenced genes and morphology

The data repository (DOI 10.17632/7vrfjhbdxs.1) contains

- Alignment files for 5 gene partitions
- Alignments for i) molecular five gene dataset (fasta) and partition file, ii) matrix for morphological data (nexus), iii) partition file to combine the molecular five gene dataset and morphological matrix in an IQ-TREE ML run
- IQ-TREE log files for the ML search for the 3 matrices
- Resulting IQ-TREE tree files for the 3 matrices

We also show the resulting tree files here color coded by major lineage for easier comparison.

Molecular five gene dataset

Molecular dataset partitioned by codon position for COI and H3. Includes model selection and ultrafast bootstrap

IQ-TREE command

```
iqtree -s DNA.fa -spp DNA.partitions.txt -m MFP -pre DNA -bb 1000
```

DNA.partitions.txt

```
DNA, p1_COI_1 = 1-514\3
DNA, p1_COI_2 = 2-515\3
DNA, p1_COI_3 = 3-516\3
DNA, p2_16S = 517-1150
DNA, p3_18S = 1151-3424
DNA, p4_28S = 3425-3832
DNA, p5_H3_1 = 3833-4157\3
DNA, p5_H3_2 = 3834-4158\3
DNA, p5_H3_3 = 3835-4159\3
```

Output DNA partitions file with the chosen best scheme (BIC) (DNA.best_scheme.nex)

```
#nexus
begin sets;
  charset p1_COI_1 = 1-514\3;
  charset p1_COI_2 = 2-515\3;
```

```
  charset p1_COI_3 = 3-516\3;
  charset p2_16S = 517-1150;
  charset p3_18S = 1151-3424;
  charset p4_28S = 3425-3832;
  charset p5_H3_1 = 3833-4157\3;
  charset p5_H3_2 = 3834-4158\3;
  charset p5_H3_3 = 3835-4159\3;
  charpartition mymodels =
    SYM+I+G4: p1_COI_1,
    TVM+F+I+G4: p1_COI_2,
    TIM2+F+ASC+G4: p1_COI_3,
    GTR+F+I+G4: p2_16S,
    TNe+I+G4: p3_18S,
    TN+F+I+G4: p4_28S,
    TIM2+F+I+G4: p5_H3_1,
    K3P+I+G4: p5_H3_2,
    GTR+F+I+G4: p5_H3_3;
end;
```

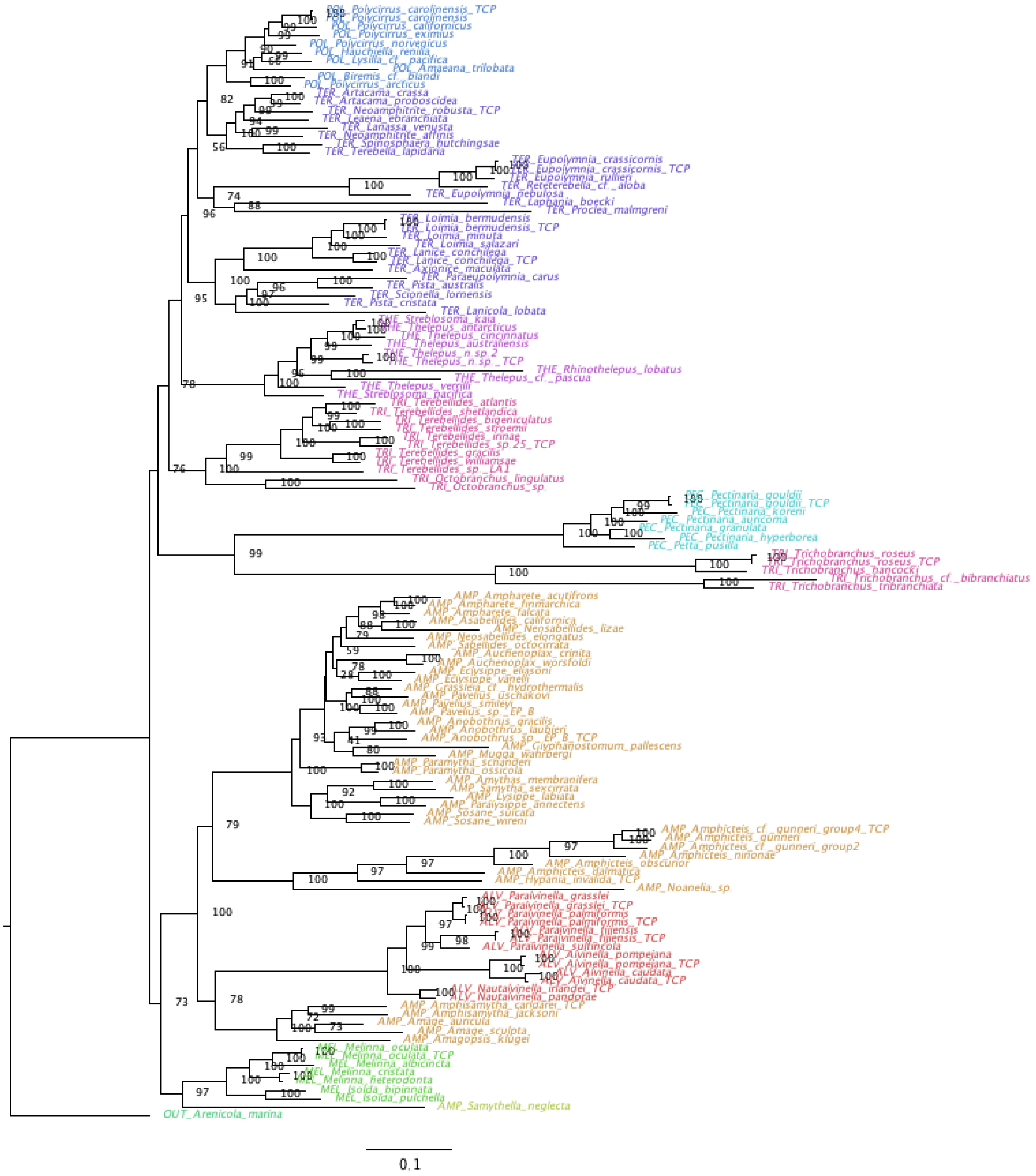

Supplementary Figure S8: ML tree for the molecular five gene dataset.

Morphological matrix

IQ-TREE command includes selection of best fit model among MK models (BIC chose MK+FQ+ASC+R3) and ultrafast bootstraps

```
iqtree -s Morphology_simplified.nex -m MFP -bb 1000 -pre morphology
```

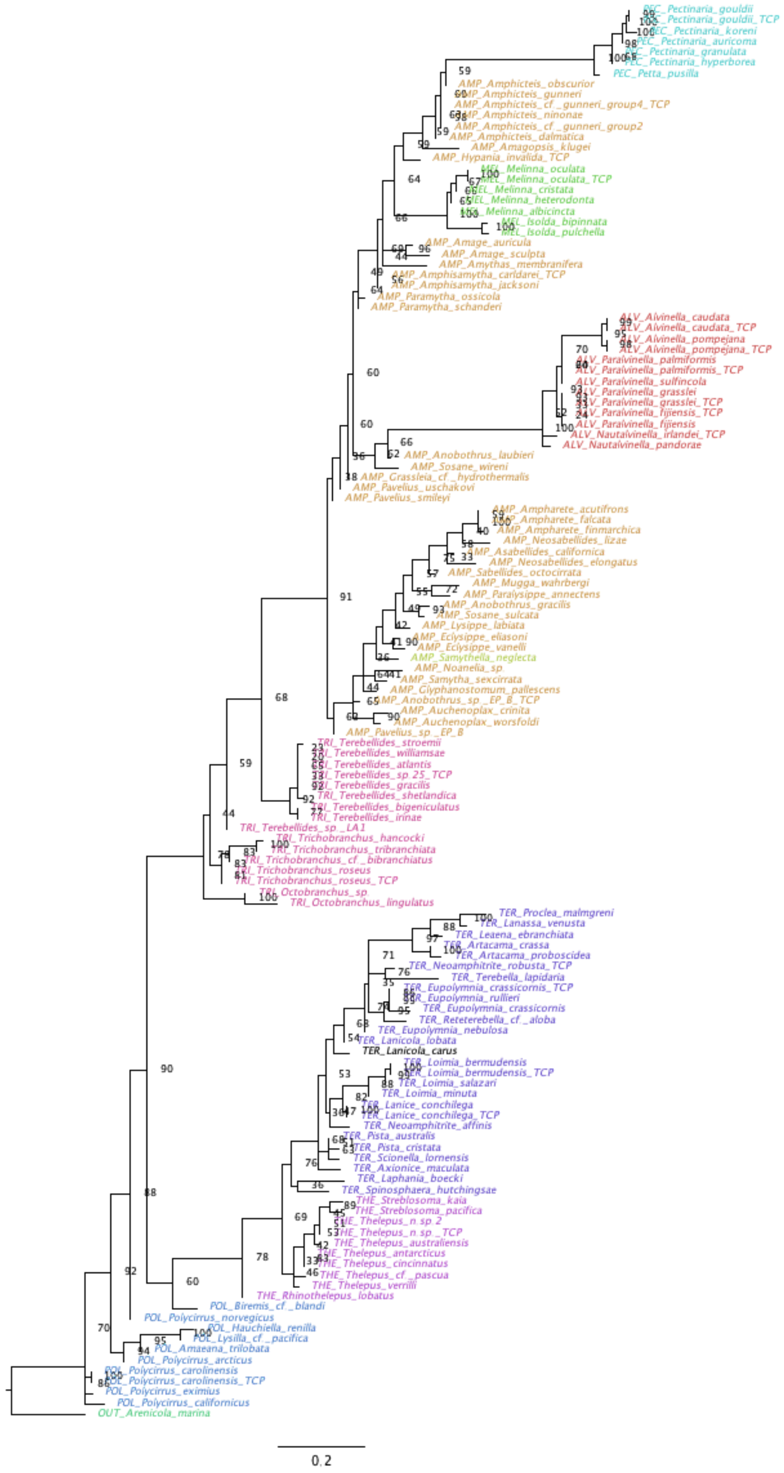

Supplementary Figure S9: ML tree for the morphological dataset based on 90 characters.

Molecular five gene matrix and morphology, unconstrained

Molecular dataset concatenated with morphological dataset, not constrained by transcriptome topology.
IQ-TREE command uses a partition file to reference alignments of different datatypes (DNA, morphological characters). Models for partitions were previously selected with ModelFinder as described above.

```
iqtree -spp combined_partitions.txt -bb 1000 -pre
combined_unconstrained
```

Partitions file (combined_partitions.txt) with the chosen best scheme (BIC)

```
#NEXUS

Begin sets;

charset p1_COI_1 = DNA.fa:1-514\3;
charset p1_COI_2 = DNA.fa:2-515\3;
charset p1_COI_3 = DNA.fa:3-516\3;
charset p2_16S = DNA.fa:517-1150;
charset p3_18S = DNA.fa:1151-3424;
charset p4_28S = DNA.fa:3425-3832;
charset p5_H3_1 = DNA.fa:3833-4157\3;
charset p5_H3_2 = DNA.fa:3834-4158\3;
charset p5_H3_3 = DNA.fa:3835-4159\3;
charset p6_morphology = Morphology_simplified.nex:1-90;
charpartition mine = SYM+I+G4:p1_COI_1, TVM+F+I+G4:p1_COI_2,
TIM2+F+ASC+G4:p1_COI_3, GTR+F+I+G4:p2_16S, TNe+I+G4:p3_18S,
TN+F+I+G4:p4_28S, TIM2+F+I+G4:p5_H3_1, K3P+I+G4:p5_H3_2,
GTR+F+I+G4:p5_H3_3, MK+FQ+ASC+R3:p6_morphology;

End;
```

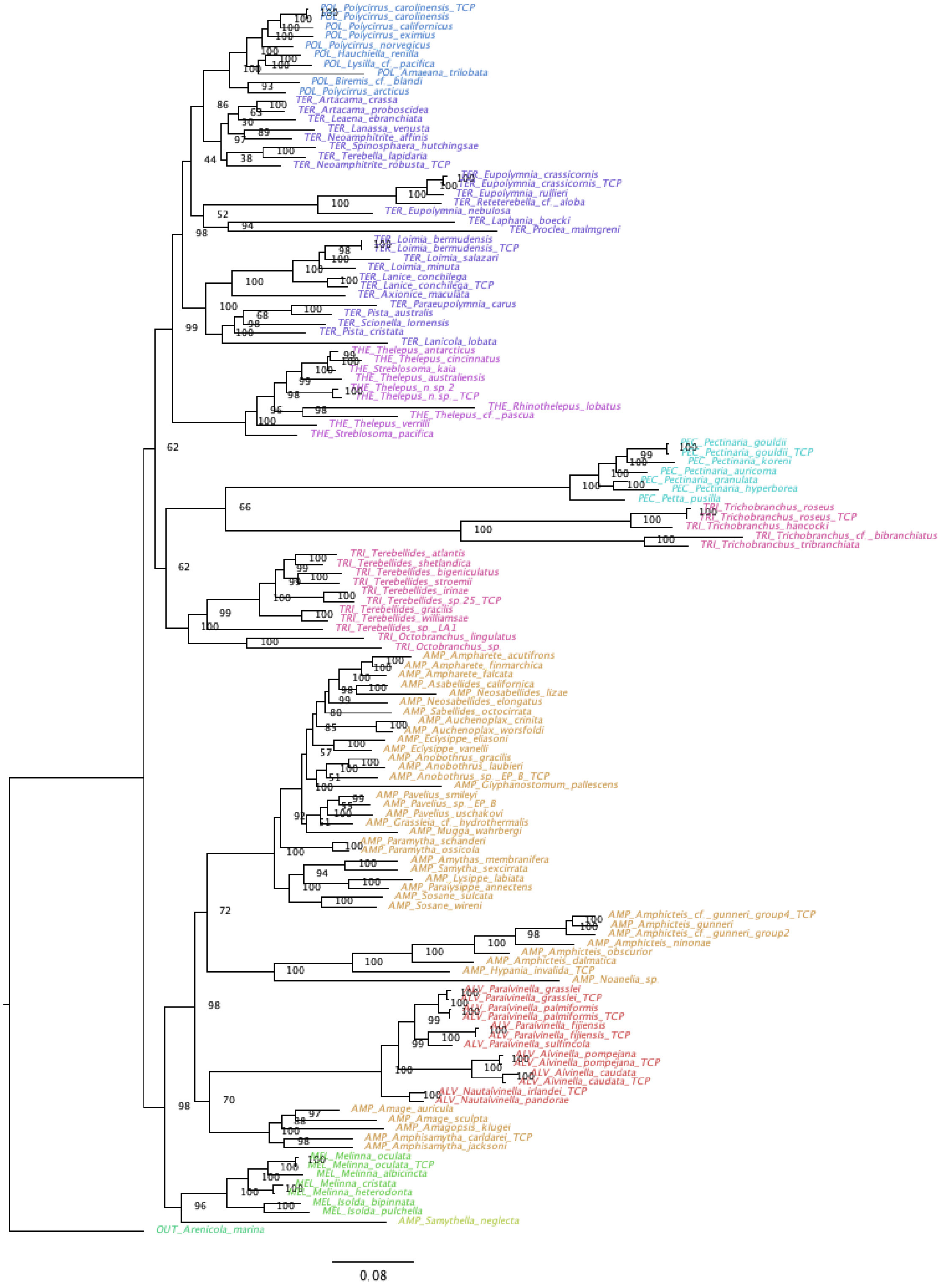

Supplementary Figure S10: ML tree for the five gene molecular matrix combined with the morphological dataset based on 90 morphological characters (unconstrained).

Molecular five gene matrix and morphology, constrained to transcriptome topology

The total evidence dataset was constrained using IQ-TREE to the backbone transcriptome topology (80% occupancy matrix).

```
iqtree -spp combined_partitions.txt -bb 1000 -g
alns_17taxa.constraint_topology.tre -pre combined_constrained
```

Partitions file (combined_partitions.txt) with the chosen best scheme (BIC)

```
#NEXUS

Begin sets;

charset p1_COI_1 = DNA.fa:1-514\3;
charset p1_COI_2 = DNA.fa:2-515\3;
charset p1_COI_3 = DNA.fa:3-516\3;
charset p2_16S = DNA.fa:517-1150;
charset p3_18S = DNA.fa:1151-3424;
charset p4_28S = DNA.fa:3425-3832;
charset p5_H3_1 = DNA.fa:3833-4157\3;
charset p5_H3_2 = DNA.fa:3834-4158\3;
charset p5_H3_3 = DNA.fa:3835-4159\3;
charset p6_morphology = Morphology_simplified.nex:1-90;
charpartition mine = SYM+I+G4:p1_COI_1, TVM+F+I+G4:p1_COI_2,
TIM2+F+ASC+G4:p1_COI_3, GTR+F+I+G4:p2_16S, TNe+I+G4:p3_18S,
TN+F+I+G4:p4_28S, TIM2+F+I+G4:p5_H3_1, K3P+I+G4:p5_H3_2,
GTR+F+I+G4:p5_H3_3, MK+FQ+ASC+R3:p6_morphology;

End;
```

The resulting tree is shown in the main text Figure 2.

Molecular five gene matrix, constrained to transcriptome topology

The molecular five gene analysis was constrained using IQ-TREE to the backbone transcriptome topology (80% occupancy matrix).

```
iqtree -sp DNA.fa -spp DNA.best_scheme.nex -bb 1000 -g
alns_17taxa.constraint_topology.tre -pre DNA_constrained
```

Partitions file (DNA.best_scheme.nex) is the partition file with the chosen best scheme (BIC) as it was morphological analysis alone

```
#nexus
begin sets;
  charset p1_COI_1 = 1-514\3;
  charset p1_COI_2 = 2-515\3;
  charset p1_COI_3 = 3-516\3;
  charset p2_16S = 517-1150;
  charset p3_18S = 1151-3424;
  charset p4_28S = 3425-3832;
  charset p5_H3_1 = 3833-4157\3;
  charset p5_H3_2 = 3834-4158\3;
  charset p5_H3_3 = 3835-4159\3;
  charpartition mymodels =
    SYM+I+G4: p1_COI_1,
    TVM+F+I+G4: p1_COI_2,
    TN+F+ASC+R4: p1_COI_3,
    GTR+F+R6: p2_16S,
    TIM2e+I+G4: p3_18S,
    TIM3+F+R4: p4_28S,
    TIM2+F+I+G4: p5_H3_1,
    K3P+I: p5_H3_2,
    GTR+F+I+G4: p5_H3_3;
end;
```

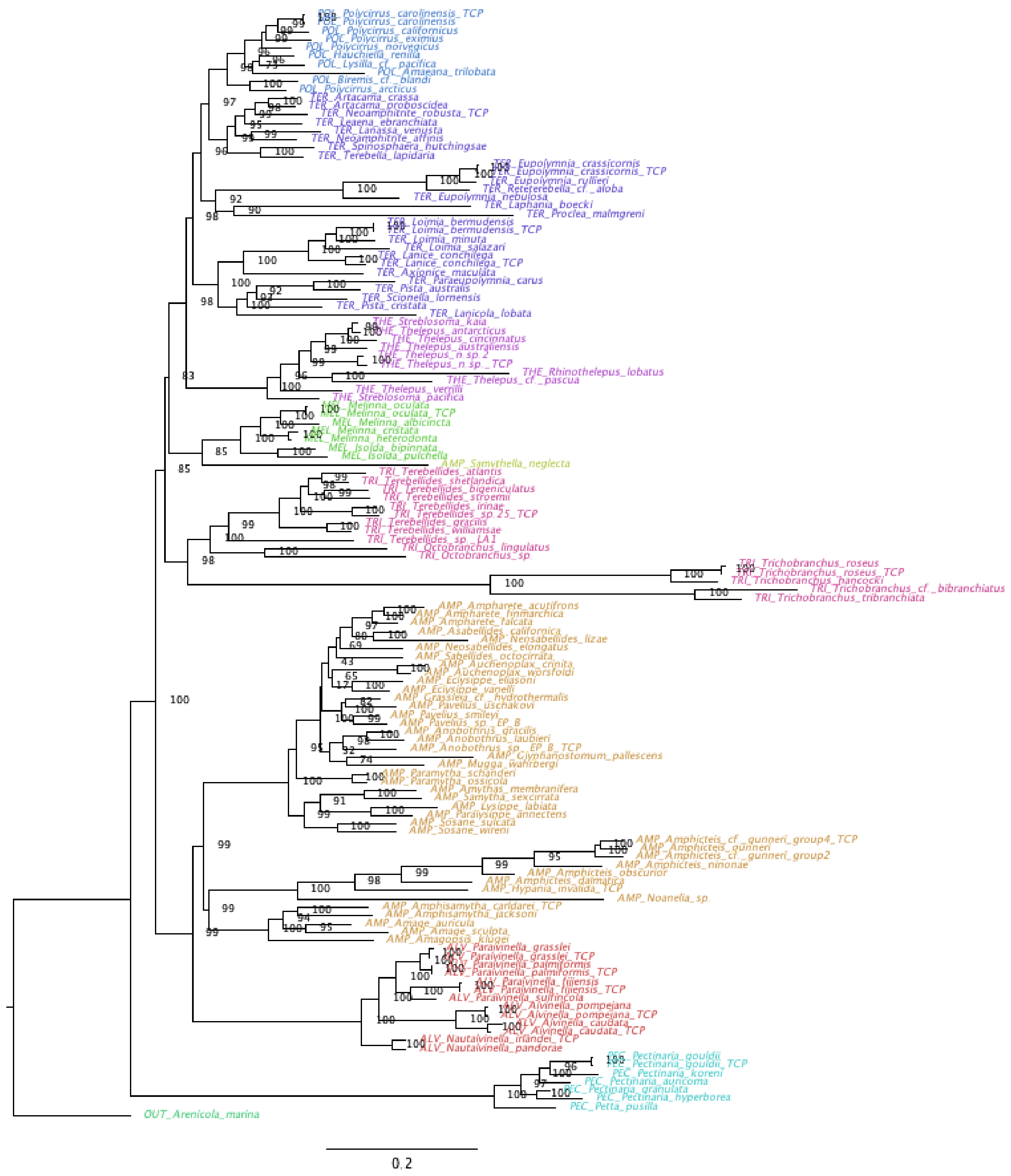

Supplementary Figure S11: ML tree for the five gene molecular matrix (without the morphological partition) constrained to the transcriptome backbone.
