# Supplementary material for: Spaghetti to a Tree: A Robust Phylogeny for Terebelliformia (Annelida) Based on Transcriptomes, Molecular and Morphological Data
Source: Biology (Basel). 2020 Apr 6;9(4):73. doi: 10.3390/biology9040073 (PMC7236012; doi:10.3390/biology9040073)
Supplement: Supplementary file 1 [file biology-09-00073-s001.zip › Supplementary file 2.pdf]

Supplementary file 2: Overview of characters and description of each state

Table of contents

Characters and character states overview.

Characters and character state description.

Body.

Head.
- Prostomium.
- Peristomium.
- Palps.

Anterior body ('thorax').
- Branchiae.
- Ventrum / glandular areas
- Segmental organs.

Parapodia and chaetae.
- Notopodia and notochaetae
- Neuropodia and neurochaetae.

Terminal part of the body.
- Pygidium.

References

Characters and character states overview.

1. Body, shape
    0 uniformly tapering
    1 anteriorly much broader

2. Prostomium, differentiation
    0 distinct from peristomium
    1 fused to peristomium (cephalic veil)

3. Prostomium, shape
    0 rounded
    1 hood-like flattened and bi-lobed
    2 expanding sheet-like over peristomial upper lip
    3 narrow ridge
    4 flattened

4. Prostomium, distal and basal parts, differentiation
    0 indistinct
    1 distinct

5. Eyespots
    0 absent
    1 present

6. Eyespots, number
    0 two
    1 more than two

7. Glandular ridges
    0 absent
    1 present

8. Peristomium, shape
    0 forms ring
    1 forms ring and upper and lower lips

9. Peristomium, upper lip, shape
    0 compact
    1 expanded and frilly

10. Inflatable front
    0 absent
    1 inflated lower lip
    2 inflated first segment

11. Multiple palps
    0 absent
    1 present

12. Palps, location in adult

0 outside mouth
1 inside mouth

13. Leaf-shaped palps
0 absent
1 present

14. Papillate palps
0 absent
1 present

15. Specialized sex palps
0 absent
1 present

16. Operculum
0 absent
1 present

17. Lateral lobes on anterior segments
0 absent
1 present

18. Lateral lobes, shape
0 raised crests
1 large lappets
2 upwards facing

19. Branchiae
0 present
1 absent

20. Branchiae, location
0 on mid to posterior body
1 on anterior segments

21. Branchiae, position relative to notopodia
0 in line or ventral
1 dorsal

22. Number of branchial pairs
0 one
1 two
2 three
3 four
4 more than 4

23. Branchiae, arrangement
0 segmental
1 crowded on anterior segments

24. Branchiae, telescoped branchiae, pattern
0 rhomboid pattern
1 horizontal transverse row

2 horizontal transverse row with one behind

25. Branchial pairs, distance
    0 dorsally adjacent or very close
    1 well separated

26. Branchial membrane
    0 absent
    1 present

27. Branchial trunk, shape
    0 digitate
    1 branching
    2 multiple filaments

28. Branchiae of successive segments, shape
    0 similar
    1 different

29. Digitate branchial trunk, shape
    0 single long filament
    1 multiple short filaments

30. Digitate trunk, secondary structures
    0 absent
    1 present

31. Digitate trunk, secondary structures, type
    0 lamellae
    1 multiple rows of simple filaments

32. Branching trunk, branching pattern
    0 dichotomous
    1 plumose

33. Building organ (cement glands)
    0 absent
    1 present

34. Ventral glandular area on anterior segments
    0 absent
    1 present

35. Ventral glandular areas, shape
    0 central glandular shield
    1 glandular stripe
    2 central shield and lateral papillae / shields

36. Segmental organs on S3
    0 absent
    1 present

37. Segmental organs on S4
    0 absent

1 present

38. Segmental organs on S5
    0 absent
    1 present

39. Segmental organs on S6
    0 absent
    1 present

40. Segmental organs on S7
    0 absent
    1 present

41. Segmental organs on S8
    0 absent
    1 present

42. Segmental organs on S9
    0 absent
    1 present

43. Segmental organs on S10
    0 absent
    1 present

44. Segmental organs on S11
    0 absent
    1 present

45. Segmental organs on S12
    0 absent
    1 present

46. Placement of pores
    0 laterally
    1 dorsally

47. Notochaetae
    0 present
    1 absent

48. Normal notochaetae starting from segment
    0 segment 2
    1 segment 3
    2 segment 4
    3 segment 5
    4 segment 6
    5 segment 7
    6 segment 8
    7 segment 9

49. Number of segments with notochaetae
    0 14

1 less than 14
2 15
3 16
4 17
5 18
6 more than 19

50. Posterior segments, parapodial elements
0 no parapodia
1 biramous
2 uniramous (abdomen)

51. Posterior segments, uniramous abdomen, notopodial rudiments
0 absent
1 present

52. Notochaetae, capillary chaetae, type between ant and post tier
0 same
1 different

53. Notochaetae, capillary chaetae, teeth or hairs
0 present
1 absent

54. Notochaetae, capillary chaetae with broad margins
0 absent
1 present

55. Notochaetae, notopodial spines
0 absent
1 present

56. Notopodial spines, type
0 projecting forward as a pair of paleae
1 one or two pairs of lateral hooks
2 a pair of dorsal hooks

57. Notopodial spines, position
0 behind the head
1 on anterior body

58. Notopodial spines on segment
0 2
1 3
2 6 and 7
3 9

59. Notopodial spines, paleae, size relative to normal capillary chaetae
0 smaller
1 larger

60. 1st notopodium, size relative to following
0 similar
1 smaller

61. Anterior notopodia, arrangement
    0 all at similar height
    1 anterior pairs displaced dorsally

62. Arrangement of the posterior notopodia
    0 all at similar height
    1 single pair displaced dorsally

63. Notopodial cirri
    0 absent
    1 present

64. Neuropodia with chaetae
    0 present
    1 absent

65. Posterior segments, neuropodial elements
    0 achaetous
    1 achaetous except one pair scaphal hooks
    2 with neurochaetae throughout

66. Neurochaetae starting from segment
    0 2
    1 5
    2 6
    3 7
    4 8
    5 9
    6 10
    7 11
    8 more than 13

67. Neuropodial cirri
    0 absent
    1 present

68. Rows of uncini, number
    0 single rows throughout
    1 double rows on some segments

69. Orientation of double rows of uncini
    0 teeth face each other
    1 teeth face away from each other

70. Start of the double rows of uncini on segment
    0 11
    1 10

71. Neuropodial tori, anterior segments, elevation from body wall
    0 low
    1 distinct lobes

72. Neuropodial tori, posterior segments, elevation from body wall
    0 low
    1 distinct lobes

73. Neurochaetae, anterior segments, type
    0 hooks
    1 uncini
    2 spines followed by uncini
    3 spines followed by hooks

74. Neurochaetae, posterior segments, type
    0 uncini
    1 spines

75. Uncini or hooks, anterior segments, capitium, organization
    0 arched rows over rostrum
    1 single tooth over rostrum
    2 single column
    3 2 columns
    4 3 columns
    5 more than 3 columns

76. Uncini, posterior segments, capitium, organization
    0 arched rows over rostrum
    1 single tooth over rostrum
    2 single column
    3 2 columns
    4 3 columns
    5 more than 3 columns

77. Uncini or hooks, rostrum
    0 present
    1 absent

78. Uncini or hooks, anterior segments, subrostrum, size relative to capitium/rostrum
    0 same or smaller
    1 larger

79. Uncini, posterior segments, subrostrum, size relative to capitium/rostrum
    0 same or smaller
    1 larger

80. Uncini or hooks, anterior segments, subrostrum, subrostral process
    0 absent
    1 present

81. Uncini, posterior segments, subrostrum, subrostral process
    0 absent
    1 present

82. Uncini or hooks, anterior segments, subrostrum, subrostral process, position
    0 near the teeth
    1 median subrostrum
    2 near anterior process

3 spanning entire subrostrum

83. Uncini, posterior segments, subrostrum, subrostral process, position
0 near the teeth
1 median subrostrum
2 near anterior process
3 spanning entire subrostrum

84. Uncini, anterior segments, anterior process, shape
0 pointed
1 rounded

85. Uncini, posterior segments, anterior process, shape
0 pointed
1 rounded

86. Hooks, beard surrounding the teeth
0 present
1 absent

87. Pygidial cirri
0 absent
1 present

88. Pygidial eyespots
0 absent
1 present

89. Life style
0 free living
1 tube-dwelling

90. Tube, consistency
0 soft
1 solid

Characters and character state description.

The systematic names used here will follow the main phylogenetic hypothesis outlined from the transcriptomic data. The homologization of the main characters is described in Appendix A.

Body.

1. Body, shape.

The outgroup, *Arenicola marina,* has a relatively uniform body diameter and tapers only slightly posteriorly in the tail region (-0). The terebelliform body is also often long and uniformly tapering (-0) as in most Thelepodinae, Terebellini, Lanicini, Trichobranchidae and Alvinellidae, and it can be straight or coiled (Figure 1). In contrast, most Polycirrinae (especially *Biremis*) and several Ampharetidae are anteriorly much broader (-1) than in the remaining body. The sturdy body of Pectinariidae represents an extreme of the latter state, where the abdominal segments are reduced to a short and thin flap, the scaphe (Figure A2D).

Head.

Prostomium.

2. Differentiation.

The prostomium is distinct from the peristomium in most Terebelliformia and the outgroup Arenicolidae (-0), but is fused with the peristomium to form the cephalic veil in Pectinariidae (-1, Figure A2c) [1]. In Polycirrini and Trichobranchidae, the tentacle-bearing prostomium is closely affiliated with the upper lip as it covers the upper lip in a sheet-like fashion (*pr,* Figure A3a, d, see character 3-2). The peristomial upper lip can still be distinguished as the distal edge of the prostomial-peristomial complex (*pe* in Figure A3D; 'free edge' in Fig. 6 of [2] and is therefore coded as distinct from the peristomium (-0).

3. Shape of the distinct prostomium (subordinate to character 2-0).

Arenicola marina has a rounded prostomium (-0). The prostomium of Ampharetidae and Melinnidae extends anteriorly and lies over the buccal cavity as a flattened hood-shaped lobe (-1, Figure A2A-B). In Alvinellidae it bears a dorsal median furrow (Figure A1c). In Polycirrini and Trichobranchidae, the distal part of the prostomium is flattened over the upper lip (-2, Figure A3A, D). In Lanicini, Terebellini, and Thelepodinae the prostomium forms a narrow transverse ridge (-3, Figure A3b) atop the base of the peristomial upper lip.

4. Differentiation of distal and basal parts of prostomium (subordinate to character 2-0).

Alvinellids have indistinguishable distal and basal parts (Figure A1C), as does the outgroup [1] (-0). Ampharetidae, Melinnidae, Terebellidae and Trichobranchidae have distinguishable regions on their prostomia (-1). The prostomium of Terebellidae consists of a basal part that often carries eyespots, and lies behind a distal part that is the origin of the buccal tentacles (Figure A3B). In Polycirrini and Trichobranchidae, this distal part is expanded (Figure A3A, D). Most Ampharetidae and Melinnidae have two prostomial lobes, a median and a U-shaped lateral one surrounding the former (Figure A2A, B). We consider these lobes homologous to the basal and distal parts of Terebellidae and adopt this terminology. Note that the prostomium in Ampharetidae is often inaccurately described as being 'trilobed' although only its anterior margin is trilobed.

5. Eyespots.

Eyespots are absent (-0) in Alvinellidae and in *Arenicola marina* [1]. The basal part of the prostomium can bear simple ocelli (-1) either located dorsally like in some ampharetids or extending laterally as seen in many terebellids (e.g. Figure 2H, A3B).

6. Number of eyespots (subordinate to character 5-1).

While *Pectinaria* and a few ampharetids have just two ocelli (-0), all other eye-bearing taxa have more than two (-1).

7. Glandular ridges.

In most taxa, the prostomium is smooth (-0), but in *Amphicteis, Hypania* and *Melinna* it bears a pair of elongate glandular ridges (-1) that diverge anteriorly (Figure 2C, A2A).

Peristomium.

8. Shape.

Arenicolids have a peristomial ring only and no lips (-0) [1]. In Ampharetidae, Alvinellidae and Melinnidae, the peristomium forms a lower lip ventrally and extends inside the buccal cavity to form the upper lip (-1, Figure A1a, b). The peristomium also forms large upper and lower lips in front of the prostomium in most terebellids and trichobranchids (-1, Figure A3D, F). This character could not be scored for Pectinariidae owing to the fusion of the peristomium and the prostomium (see character 2-1).

9. Upper lip, shape (subordinate to character 8-1).

The upper lip can be compact and small (-0) as in Ampharetidae, Alvinellidae, Melinnidae and most Terebellidae. In Polycirrini, the thelepodin *Rhinothelepus lobatus* and Trichobranchidae, it is expanded and frilly (-1) and usually folded into three lobes (Figure A3A, D). When viewed from above, the upper lip of

Polycirrini and Trichobranchidae is just a narrow band, almost entirely covered by the prostomium (cf. character 3-2). In *Rhinothelepus lobatus* the upper lip is similarly expanded and folded (see fig. 6 in [3]), but not covered by the prostomium. This large upper lip was the proposed autapomorphy of Telothelepodidae sensu [4] but is shown here to be a secondary expansion within Thelepodinae.

10. Inflatable front.

A true proboscis is present in *Arenicola marina*, which is everted from within the buccal cavity and maybe used for inflating the anterior end for burrowing, as well as feeding [5]. *Arenicola* lacks any other inflationary mechanism, and most other taxa in this study also lack such structures (-0). However, in the Terebellini *Artacama* and certain species of *Trichobranchus* (Figure A3E, F) can inflate the ventral body wall near the head as what is often called a 'proboscis'. *Trichobranchus* species have a rugose first segment that can be inflated to certain degrees, forming a distinct inflatable 'proboscis' as in *T. tribranchiata* (Figure A3E) and *T. hancocki*. Originally, taxa with such a proboscis were separated in *Artacamella* and thought to be related to the terebellin *Artacama* [6], but later studies showed that *Artacamella* was part of Trichobranchidae and a junior synonym of *Trichobranchus* [7–10]. We agree with [3] that the probosci of *Artacama* and *Trichobranchus* are formed by different structures; by the peristomial lower lip in *Artacama*, and by the first segment in *Trichobranchus*

(compare Figure A3E and F). In *Artacama*, the papillated proboscis (-1, *pe* in Figure A3F) has a loop of the oesophagus in it [11], plate 6, figure 1 in [12]). We do not agree that the proboscis is the first segment [13,14], as this segment clearly lies behind the proboscis and has a smoother texture (*I* in Figure A3F; [3,7,15]. In *Trichobranchus tribranchiata* and *T. hancocki*, however, the proboscis does appear to be part of the first segment (-2) (*I* in Figure A3E) [2,7,8], contra [10]. Here, the peristomial lower lip is similar to other Trichobranchidae, and is present between the mouth opening and the proboscis [3].

Palps.

11. Multiple palps (buccal tentacles).

Palps are absent in Arenicolidae (-0). Terebelliforms possess multiple food-gathering appendages (buccal tentacles) that are grooved, U-shaped in cross-section, and possess a longitudinal row of cilia (-1) [16,17]

12. Location of palps in adults (subordinate to character 11-1).

Most terebelliforms cannot retract the palps into the mouth and they are therefore prostomial and permanently exposed (-0). The palps of Ampharetidae, Alvinellidae and Melinnidae originate inside of the buccal cavity, are retractable and are arguably peristomial (-1, see discussion above). Palps of Pectinariidae are located outside of the mouth (-2) and are thought not to be retractable.

13. Leaf-shaped palps (subordinate to character 11-1).

Most terebelliforms have cylindrical palps of the same diameter throughout (-0). Distally inflated, leaf-shaped palps (-1) can be the only type of palp present (e.g. *Octobranchus lingulatus*), or they can be present in addition to the common cylindrical ones (as in many Polycirrini, and *Trichobranchus*).

14. Papillate palps (subordinate to character 11-1).

Palps are usually smooth (-0) but the amparetids *Ampharete*, *Asabellides*, *Neosabellides* and *Sabellides* have pinnate palps (-1).

15. Specialized sex palps (subordinate to character 11-1).

Palps are usually not known to be involved in reproduction (-0), but alvinellids show palp dimorphism in relation to the sex (-1). In males, enlarged palps seem to be involved in sperm transfer [18–21].

Anterior body ('thorax').

16. Operculum.

In the outgroup and most terebelliforms, the second and third segments are similar to the following ones and not fused (-0). Pectinariidae have an

anterodorsal plug, the operculum (-1), which consists of two fused segments (Figure A2C, D).

17. Lateral lobes on anterior segments.

Lateral lobes are protrusions of the body wall of the anterior segments. Such lobes are absent in *Arenicola*, Ampharetidae, Alvinellidae and Thelepodinae (-0). They are present in many Terebellidae (Figure 2H) and in some Trichobranchidae (-1). We consider the ventro-lateral collar of Melinnidae a lateral lobe (-1, Figure 2E, A2A).

18. Shape of the lateral lobes (subordinate to character 17-1).

In Melinnidae, the first five segments are united to form a continuous upwards-facing collar (-2, Figure 2E, Figure A2A) that conceals the basal part of the branchiae. Similar collar-like lappets (-2) covering the branchiae are present in *Octobranchus lingulatus* [22] and "Bizzarobranchus" (SCAMIT newsletter), where the lappets project forward on the ventral side and laterally towards the brachiae. In many Terebellinae, lateral lobes may be present as thickened regions along the anterior lateral margins of the segment (e.g. *Eupolymnia* species but not in *Reteterebella alobata*) that only slightly project forward, forming raised crests (-0). In all Lanicini included here, the lobes are larger, often rounded, triangular or collar-like, and project anteriorly covering much of the preceding areas (-1,

Figure A2g).

Branchiae.

19. Branchiae

Branchiae are present in most Terebelliformia and the outgroup (-0) but are absent in some Terebellinae and Thelepodinae (-1). A few unsampled species of Ampharetidae and Pectinariidae also lack branchiae [23]. Within Terebellinae, they are absent in all Polycirrini, and in *Laphania boecki*, *Proclea malmgreni*, as well as some Terebellini (*Lanassa venusta, Leaena abranchiata*, *Spinosphaera hutchingsae*). Our specimen of the thelepodin *Streblosoma kaia* had completely reduced branchiae, but this species can have rudimentary papillae in place of the branchiae [24].

20. Location (subordinate to character 19-1).

Arenicola marina has parapodial branchiae further posterior on the mid- to posterior parts of the body (-0). In terebelliforms, the branchial pairs are located on the anterior segments behind the head and the first segment (-1), starting from segment II.

21. Position relative to notopodia (subordinate to character 19-1).

The branchiae of most terebelliforms are located dorsally, above the notopodia (-1). In Pectinariidae they are inserted laterally, on the level or below the line of notopodia (-0). This state is also found in the first pair of filamentous branchiae of some *Thelepus* species (e.g. *Thelepus* n.sp. Figure 1, *T. antarcticus,* Figure A3b) and *Rhinothelepus lobatus* [25] that form a half ring extending downwards below the notopodial line.

22. Number of branchial pairs (subordinate to character 19-1).

The number of branchial pairs in Terebellidae varies from three (-2), two (-1, e.g. *Pista cristata*, *Lanicola lobata*) or one (e.g. *Axionice maculata*, -0). Only one terminal included here (*Streblosoma pacifica* (Thelepodinae), among a few other unsampled ones) has more than four pairs of branchiae (-4), which has filamentous branchiae from segment II, extending to segment VI or VII [26]. In Trichobranchidae, two to four pairs of branchiae exist. The single fused branchial stalk of *Terebellides* is interpreted as consisting of two pairs of branchiae (-1), which are fused [3]. It is noted that [27] suggest a possible third, rudimentary branchial pair. Most Ampharetidae have four pairs of branchiae (-3), but can have two or three pairs (e.g. *Samytha sexcirrata, Mugga wahrbergi, Eclysippe vanelli* and *Auchenoplax crinita*). Alvinellidae, Pectinariidae and most Melinnidae have four pairs (-3).

23. Arrangement of branchiae (subordinate to character 19-1).

In most terebelliforms with multiple pairs of branchiae, they are segmentally arranged on the anterior segments of the thorax (-0). In adult Ampharetidae, Alvinellidae and Melinnidae, up to four pairs of branchiae appear clustered anteriorly due to branchial migration during early ontogeny (see above), and thus appear to be located on only one to two segments (-1).

24. Pattern of the grouped branchiae (subordinate to character 23-1).

The clustered (sometimes called telescoped) branchiae of ampharetids, alvinellids and melinnids can be arranged in several configurations. The most common arrangement found in Alvinellidae, Melinnidae, and in some Ampharetidae (e.g. *Amphisamytha, Amphicteis*) is a rhomboid pattern with a 2+2 or a 1+2+1 configuration (-0). The most extreme form of anterior displacement results in all branchiae being arranged in a transverse row on a single segment (-1, *Auchenoplax crinita, Eclysippe vanelli*, *Samytha sexcirrata* Figure A2B). This arrangement only occurs in species with less than four branchial pairs. In those with three or four pairs of branchiae, two or three pairs can be arranged in a horizontal line, with the third or fourth pair slightly displaced behind this row, resulting in a 2+1 or 3+1 arrangement (-2, e.g. *Ampharete* species, *Sabellides octocirrata, Mugga wahrwergi*).

25. Distance of the branchial pairs (subordinate to character 19-1).

The paired branchiae on each side of the body can be either well separated (-1) or can be medially so close to each other that a gap between the branchial pairs is missing as (-0) as in Melinnidae and Alvinellidae (Fig A1C). Ampharetids have both states. The median median fusion of branchial pairs in *Terebellides* is considered as an extreme case of state -0.

26. Branchial membrane (subordinate to character 19-1).

The basal parts of the branchia inserting on the dorsal body wall may be separate from one another (-0) or united by a basal branchial membrane (-1) as seen in several ampharetids. The latter state is particularly pronounced in Melinnidae, where the branchial pairs are basally fused to varying degrees (Figyre 2e). Due to the fusion, the branchiae appear to be grouped on a transverse branchial ridge, which partially overhangs the segments behind the head. We also consider the sheath uniting the fused branchiae of *Terebellides* as a branchial membrane (-1).

27. Shape of branchial trunk (subordinate to character 19-1).

Each branchia is composed of a main trunk that can either be an unbranched digitate filament (-0), or split up into several branches (-1). We interpret several complex branchial structures as being derived from digitate filaments and not as branching:

Digitate filaments are present in all ampharetids, alvinellids and melinnids (e.g. Figure 2b-e, Figure A2A-B). All included Ampharetidae and most Melinnidae have unbranched filaments, but *Isolda bipinnata* (Melinnidae) has feathery lamellae emerging from the trunk (Figure 2e) as do unsampled Ampharetidae such as *Phyllocomus hiltoni*. We do not consider those to be branches of the main trunk, but secondary structures (see character 32). Similarly, we treat the branchiae of Alvinellidae as having unbranched main trunks, similar to the ampharetid type, which then bud off secondary structures (Figure A1C). Pectinariidae have two pairs of simple filaments ('tentacular cirri', Figure A2B), followed by two pairs of filamentous branchiae with secondary structures (*br3* and *br4* in Figure A2B). The fused branchiae of *Terebellides* species were scored as a digitate trunk with secondary structures, the four lamellar lobes (*br1+2* in Figure A3D).

In living animals, one can observe the major blood vessels running along the main trunk (e.g. Figure A2A,B, Figure A3E). These then bud smaller vessels off into the secondary structures, if present. This state is different from the branching branchiae in some Terebellinae (-1), where the main trunk itself splits up in a dichotomous or arborescent fashion, including the major vessels (e.g. *Loimia minuta* Figure 2h).

The multiple short digitate filaments (cf. character 31) of thelepodins and the Terebellini *Artacama* (Figure 2I, A3F) originate directly from the body wall. This state was scored as a digitate branchial stem (-0). An alternative explanation

for this condition is that it is derived from a branching trunk that is reduced in size so that only the terminal filaments persist. Evidence for this is that in some members of *Thelepus*, the filaments originate from a slightly protruding flap of the dorsal body wall that could be interpreted as a reduced form of a stem (see figure in [23]).

28. Shape of branchiae on successive segments (subordinate to character 19-1).
Most terebelliforms have the same branchial type (i.e. either digitate or branching) on successive segments (-0), even though the posterior pairs often decrease in size. Other taxa display different shapes (-1): In Pectinariidae, segment II and III carry filamentous branchiae (tentacular cirri), while segments IV and V have lamellate branchiae (Figure A2c). In Melinnidae, *Isolda* has two pairs pinnate and two pairs of smooth branchiae (Figure 2e). Several members of *Octobranchus* can display different shapes of branchiae on successive segments (see figure 1D-H and table 1 in [28], table 2 in [29]), but the included *O. lingulatus* has the same shape of branchiae (digitate branchiae with a thickened basal part and terminally with a long filiform process) and is therefore scored as -0. The unnamed “Bizzarobranchus” (SCAMIT newsletter) has one pair with a thickened basal part, followed by three pairs of short and thick branchiae (-1).

29. Number of digitate filaments (subordinate to character 27-0).

When digitate branchiae are present, they can be a single thick, long filament (-0, e.g. Figure 2b, c). Each branchial pair of many Thelepodinae and *Artacama* (Terebellini) consists of multiple simple filaments (-1) that are thin, short and often spirally curled (Figure 1, 2i, A3b, f).

30. Secondary branchial structures on digitate trunk (subordinate to character 27-0).

When digitate branchial stems are present, they can be smooth filaments without any lateral protuberances (-0). Other taxa develop secondary structures that bud off the main cylindrical trunk (-1) as discussed above (character 28).

31. Type of secondary branchial structures on the digitate trunk (subordinate to character 30-1).

The melinnid *Isolda* has bipinnate branchiae with lamella-like protuberances (-0, Figure 2e) arranged in two lateral rows. *Alvinella* has two series of flattened and sickle-shaped lamellae in two opposing rows (-0, Figure A1B). In *Paralvinella*, the main trunk carries multiple rows of densely packed filaments (-1, Figure A1A, C).

In *Terebellides*, each lobe carries a series of thin semicircular lamellae (-0, Figure 2K, A3D), which are similar to, but more abundant than the lamellae on the posterior branchiae of Pectinariidae (-0, Figure 2A, A2C). The undescribed

species *Terebellides* sp. LA1 has thick lamellae that are more distantly spaced (Figure A3c) than those of other *Terebellides.*

32. Branching pattern of branched branchiae (subordinate to character 27-1).

If a branching trunk is present, it can either split dichotomously with each branch splitting into two, usually thinner and shorter, branches (-0, Figure 2h). The branching can also be tighter spaced with the main trunk branching off numerous filaments in a crowded arrangement giving the branchiae a bottlebrush-like appearance (-1) as in *Pista cristata* and *Scionella lornensis.*

Ventrum / glandular areas

33. Building organ (cement glands).

Pectinariidae have a mid-ventral structure below the tentacles known as the 'cementing organ' [30], or 'building organ' [31] (-1), which is involved in tube building. It stains in a similar way with methyl green as the glandular stripes of Pectinariidae (personal observation) and contains large glandular cells [32]. Other taxa lack this organ (-0).

34. Ventral glandular areas on anterior segments.

All terebelliforms possess glandular areas (-1), while *Arenicola marina* and other polychaetes in general lack these (-0). We do not agree that *Biremis* cf. *blandi* (Polycirrini) lacks glandular areas [33,34]. Here, the area between the midventral ridge and the uncinigerous tori is conspicuously enlarged and reminds of the situation in other Polycirrini.

35. Shape of the ventral glandular areas (subordinate to character 34-1).

The glandular shields of Terebellini, Lanicini, *Eupolymnia, Proclea* and *Laphania* and Thelepodinae are unpaired, discrete, and significantly swollen (-0, Figure 2h, i, A3b, f). They are often trapezoidal, rectangular, or crescent-shaped and can decrease in width posteriorly. In Thelepodinae, the glandular shields are extensive, often irregularly sulcated, and may extend laterally up to the notopodia (Figure 2h, A3b). All other terebelliformis have transverse glandular stripes (-1) across their ventral sides, mostly associated with the anterior margins of the segments. In ampharetids, alvinellids and melinnids they are moderately swollen areas between the tori (Figure A2a-b). They can be better distinguished in live animals than in fixed specimens but stain with methyl green. They often extend to the tori, which can be similarly glandular, but are clearly distinct from them by a furrow. Pectinariids have distinctly swollen anterior margins on a variable number of anterior segments (Figure A2c). The glandular stripes have furrows in some species. In Trichobranchidae, the glandular stripes are also

mostly located on the anterior margin of anterior segments (Figure A3D-E), but some segments are staining completely with methyl green [35,36]. In Polycirrini, two states are present. *Hauchiella renilla* and *Lysilla* cf. *pacifica* have a central shield similar to the one in Terebellinae (-0), except that it is reduced in size and fits into the deep midventral groove. In *Amaeana trilobata* and the included *Polycirrus* species, the median shield is present in addition to conspicuous, swollen structures on the sides that can be papillate (-2, Figure A2a; [32] called these structures 'Seitenpolster').

Segmental organs.

36-45. Segmental organs on segments 3 to 12.

In annelids in general there are segmental organs along the body, though in the outgroup *Arenicola marina* these are restricted to a small number or anterior pairs from segment V to segment XI [37]. In the terebelliforms sampled here the segmental organs are never present in the first two segments, and posteriorly never behind segment XII. Of the taxa included here, the highest number of nephridia is present in *Hauchiella renilla* (10 pairs on segments III to XII). The lowest number is found with only two pairs of segmental organs, for example in alvinellins, where one pair is excretory and the other is genital [19]. Most terebelliforms have a few pairs of segmental organs in the anterior segments, for

example never more than four pairs in all Ampharetidae examined, while several Polycirrinae and *Terebella lapidaria* can have higher numbers of segmental organs up to segment XII.

46. Placement of nephridiopores.

In most annelids, the outgroup and most of the ingroup, the segmental organs mostly open in proximity to the parapodia, either between the rami or in line or slightly dorsally of the notopodia (-0). In some ampharetids and alvinellids, the opening of the anteriormost pair of segmental organs is dorsally displaced and close to the branchiae (-1). In other ampharetids (e.g. *Anobothrus gracilis*) the the segmental organs on each side of a segment open on a joint papilla between the branchiae [32,38]. Alvinellids have a joint papilla opening between the posterior branchial pair [19] that is the opening of the female spermathecae and the male seminal vesicles [39].

Parapodia and chaetae.

Notopodia and notochaetae

47. Notopodia with notochaetae.

The outgroup and most terebelliforms possess notopodia with notochaetae on their bodies (-0) but a complete absence of notochaetae is found in the Polycirrini *Hauchiella renilla* (Figure A3a) and *Biremis* cf. *blandi* (-1).

48. First normal notochaetae on segment.

The outgroup and all terebelliforms lack chaetae on segment I. We consider the notopodial spines on the second segment present in certain ampharetids, melinnids and pectinariids as distinct from normal notochaetae (see character 55) and therefore do not count these as the first notochaetae . Normal notochaetae start from segment II (-0) in *Streblosoma* species (Thelepodinae) and the outgroup *Arenicola*, and from segment III (-1) in *Thelepus, Laphania*, *Terebellides stroemii*, Polycirrini, Alvinellidae and several Ampharetidae. Segment IV carries the first notochaetae (-2) in some ampharetids, Melinnidae, *Octobranchus* and most Terebellinae (Figure A2a, B, S3F). Segment V has the first notochaetae (-3) in *Reteterebella* cf. *alobata* and "*Bizzarobranchus*". Capillary chaetae of Pectinariidae and *Trichobranchus* start from segment VI (-4, Figure A2d, S3E).

49. Number of segments with notochaetae.

Arenicola has 14 segments carrying notopodia (-0). The number of segments with notopodia is of principal importance in the taxonomy in ampharetids, melinnids, trichobranchids, pectinariids and most terebellids (Fauchald 1977; [40–44]. Of the

sampled taxa, Polycirrini and Ampharetidae have the smallest number of 'thoracic' chaetigers (e.g. 9 in *Lysilla* cf. *pacifica*, 13 in *Mugga wahrbergi*), while several dozen can be present (e.g. *Terebella lapidaria*). Less than 14 notochaetigers (-1) are relatively rare, while 14 to 18 chaetigers occur across a range of taxa (-1 through -5). Certain segment numbers are particularly common, especially 17 chaetigers (-4; [23]. Above 19 chaetigers (-6), the number is more variable.

50. Posterior segments, parapodial elements.

In the outgroup *Arenicola* the posterior body is a segmented achaetous 'tail' that lacks any parapodial elements (-0). In Alvinellidae, several Thelepodinae (e.g. *Streblosoma kaia*) and Terebellinae (e.g. *Terebella lapidaria*), the notochaetae in the posterior body continue to the end of the body (-1) and are progressively reduced in size. However, in many terebelliforms the notopodia are virtually absent, leaving only the neuropodial ramus fully developed (-2). This uniramous region is traditionally referred to as the abdomen (see above) and is present in Ampharetidae, Melinnidae, Trichobranchidae and Pectinariidae (here called the scaphe, see above). Certain Ampharetidae (e.g. *Amphicteis*) and Melinnidae lack notochaetae on posterior segments, while keeping the notopodial lobe (as notopodial rudiments, cf. character 51), which still allows for the designation of an abdomen and is scored as state -2.

51. Posterior segments, uniramous or with reduced notochaetae, notopodial rudiments (subordinate to character 50-1).

In Alvinellidae and some Terebellidae, notopodia with chaetae are developed throughout the entire length almost to the pygidium (-0). As mentioned above, melinnids and certain ampharetids and have only the notopodial shafts persist while the notochaetae are reduced, forming 'notopodial rudiments' (-1, Figure A2a) [45]. If present in the anterior body, notopodial cirri (cf. character 63) also persist on the rudiments (e.g. *Amphicteis* species).

52. Capillary chaetae, type between anterior and posterior tier.

The outgroup showstwo rows of capillary chaetae that are essentially similar (-0) [46], though the anterior one is usually being shorter than the posterior ones [3]. In terebelliforms, the two tiers can also be similar (-0) or carry different types of chaetae (-1), either in the surface structure of the capillaries (cf. character 53) or the wing development (cf. character 54). *Polycirrus* cf. *carolinensis* for example has an anterior tier of pinnate chaetae and the posterior tier of smooth chaetae. All Pectinariidae represented here have smooth capillary chaete in addition to geniculate or incised notochaetae [47,48]. The capillaries of Alvinellidae differ between tiers (*Alvinella*) or are the same (*Paralvinella*, Figure A1F).

53. Capillary chaetae (at least one tier) with teeth or hairs.

Fine hairs or teeth on the surface of the capillary chaetae (-0) are found in the outgroup *Arenicola marina [49]*, many other annelids and several terebelliforms (e.g. *Alvinella*, Figure A1f). Most terebelliforms have smooth capillaries (-1). If the anterior and posterior tiers carry both types of chaetae (cf. character 52-1), we scored the terminal with state -0.

55. Capillary chaetae (at least one tier) with broad margins.

Capillary chaetae can have no or narrow wings (-0) as in the outgroup [49]. Wings are defined as broad (-1) if the wing is wider than the main body of the chaeta (-1). The wings can be present along most or the entire length of the capillary, or be restricted to the base or the terminal part. If the anterior and posterior tiers carry both types of chaetae (cf. character 52-1), we scored as state -1.

55. Notopodial spines.

Notopodial spines are thick acicular chaetae that can be straight or curved that are present in Alvinelllidae, Pectinariidae and several Ampharetidae (-1). All other taxa lack spines (-0).

56. Type of notopodial spines (subordinate to character 55-1).

Three types of notopodial spines are recognized within Terebelliformia:

(-0) Paleae are the broad, somewhat flattened notopodial spines on segment II in pectinariids and some ampharetids that can have a metallic sheen (Figure 2a,c). They may have an opercular function or be used for digging (especially in Pectinariidae, Figure A2c-d; [23,30]). They can be thick (e.g. *Amphicteis*, Figure 2c) while in others they resemble the usual capillary chaetae (e.g. *Samytha sexcirrata*, arrow in Figure A2b; cf. character 59). The size of paleae can vary even in closely related species [50].

(-1) Alvinellids have one or two pairs of straight lateral spines interspersed normal capillary chaetae (Figure A1B,D) that may be used in territorial behavior [51]. In spite of the difference in segmental position (cf. character 57, see discussion in Appendix A), we consider them homologous in *Alvinella* and *Paralvinella* based on their morphological similarity (as [23,52]).

(-2) Melinnidae have nuchal spines on the lateral collar behind the branchiae. They are thick, more or less distally curved spines with a central canal. All taxa included here have one pair, but two pairs are found in other melinnids, or they may be absent altogether [26,53]. A neuropodial origin was proposed [23] but we view them as notopodial owing to their dorsal position and their presence alongside a neuropodial ramus on the same segment. It may be advisable to call them 'nuchal spines', not hooks (e.g. [40,54]), to reserve the latter term for neuropodial structures (see section below).

57. Position of notopodial spines (subordinate to character 55-1).

The spines are either located on the first few segments behind the head before normal capillary chaetae commence (-0) or are shifted further backwards, between segments with normal capillary chaetae, as in Alvinellidae (-1).

58. Notopodial spines on segment (subordinate to character 55-1).

The segmental position of the notopodial spines (paleae) in Ampharetidae is disputed, with proposals that they lie as on the first [3,4], second [32,40,54], or third [23,26,45,50] segment. We view them on segment II (-0, Figure A2b). The dorsal spines of Melinnidae are located one segment behind the paleae, i.e. segment III (-1; contra segment IV [45]). They are therefore positionally not homologous [54]. The position of the stout paleae in Pectinariidae is uncertain [1] but their innervation suggests that they are positionally homologous to the paleae of Ampharetidae [54]. As outlined above, this leads us to code them on segment II (-0). Based on our enumeration system of alvinellid segments (see above), the stout lateral spines are present on segment VI and VII in *Alvinella* (-2), and on segment IX in *Paralvinella* (-3).

59. Size of the paleae relative to normal capillary chaetae (subordinate to character 56-0).

Paleae are larger and stouter than the normal chaetae (-1) in all Pectinariidae and in some Ampharetidae but can be of similar or smaller size in other Ampharetidae (-0).

60. Size of the first notopodium relative to the following ones.

The first normal capillary chaetae can be of similar size to those in following segment in the outgroup and most Terebellidae (-0). In others, the first normal capillary chaetae can be smaller in size as those following (-1, Figure A2b).

61. Arrangement of anterior notopodia.

The first pair or pairs of notopodia can be at similar heights to the following (-0) as in the outgroup and most terebellids, or displaced dorsally (-1) and therefore close to the branchial bases as in Alvinellidae (Figure A1c) and many Ampharetidae.

62. Arrangement of the posterior notopodia.

The outgroup and most terebelliforms have notopodia that are arranged at a similar height throughout (-0). Exceptions are found among some ampharetid taxa, where a single notopodium in the posterior thorax is dorsally displaced (-1) and may carry unusual capillaries [50,55]. This unusual notopodium may aerate the tube [23]. For the taxa scored with this state it should be noted that they occur on different chaetigers. *Anobothrus* has the 11th notopodium elevated and the tips of the notochaetae are minutely spinulose (Figure 1; [41]). In *Mugga* the last notopodium is elevated so that the short, flattened, and frayed capillaries are

crossing middorsally. In *Sosane*, the plumose capillaries of 13th notopodium almost meet dorsally [41,50].

63. Notopodial cirri.

Notopodia are mostly smooth conical projections (-0) but in some taxa, they can carry terminal papillae or cirri as in *Amphicteis* and *Paralvinella* (-1, Figure A1D).

Neuropodia and neurochaetae.

64. Neuropodia with neurochaetae.

Neuropodia with neurochaetae are present (-0) in all taxa but *Hauchiella renilla* and *Lysilla* cf. *pacifica* (-1) which lack neurochaetae throughout their entire body. The following characters referring to neuropodia are inapplicable (-) in these species.

65. Posterior segments, neuropodial elements.

The outgroup *Arenicola marina* has a tail region that lacks chaetae (-0), characters referring to neurochaetae in the posterior body are therefore coded as inapplicable (-). The posterior segments of Pectinariidae forming the scaphe are achaetous except for one pair of neuropodial spines on the first scaphal segment (-1). Other terebelliforms have neurochaetae throughout their posterior bodies (-2).

66. First neurochaetae on segment.

Neuropodia of *Arenicola marina* start on segment II as do the aciculae of Melinnidae (-0, Figure A2A). Following the previously outlined enumeration of anterior segments, neuropodia are present from segment V in most Terebellinae (-1). Ampharetidae have uncini starting from segment VI (Figure A2B), as the hooks of *Trichobranchus* (-2, Figure A3E). Neurochaetae start from segment 9 in Pectinariidae (-5). The first uncinigers vary in Alvinellidae and other Trichobranchidae (states -3 to -8).

67. Neuropodial cirri.

There are no neuropodial cirri in the outgroup and in most of the ingroup (-0). In some species, the neuropodial tori can carry a superior papilla above the row of uncini (-1). If present, they often get more differentiated towards the posterior the long cirriform projections on the abdomen of *Sabellides octocirrata* like the large leaf-like structures like on the abdominal tori of *Artacama* species (Figure A3F).

68. Number of rows of uncini.

The outgroup and most terebelliforms have tori with a single row of hooks or uncini (-0). Only members of Terebellinae, but excluding Polycirrini, have double rows of uncini on certain segments of the thorax (-1). The double rows usually

start on segment XI and continue until the thorax ends (Figure A3f). In taxa with high numbers of notopodia (e.g. *Terebella lapidaria*), the double rows can continue beyond segment 20. In larval terebellins, the double rows originate by inversion of some of the uncini from a single row [14,56–58].

69. Orientation of the double rows of uncini (subordinate to character 69-1).
When double rows of uncini are present, they are usually oriented in a way that the rostra face each other (-0, 'beak-to-beak' of [3]). Species of *Lanice* and *Loimia* differ in that the teeth face away from each other (-1). [3] point out that the uncini of *Loimia* are 'crest-to-crest' rather than 'back-to-back' as in *Lanice*. We do not consider these different orientations of the uncini as these could arise from fixation of the muscles that move the uncini. It is possible that these slight differences can only be seen in SEM imaging, which we did not employ here.

70. Start of the double rows of uncini on segment (subordinate to character 69-1).
When double rows of uncini are present, they usually start on segment XI (-0). Members of *Eupolymnia* are special since their double rows can start from different segments than in others, namely segment X to XII (see table 1 in [59]), which has also been described for *Reteterebella* [60]. Of the species sampled here, *Eupolymnia crassicornis*, *E. rullieri* and *Reteterebella* cf. *aloba* share the double rows from segment X (-1), while their sister group *E. nebulosa* has double rows from segment XI (-0) like all other Terebellinae with uncini in double rows.

71. Anterior neuropodia tori, elevation from body wall.

The tori of the anterior body are usually low sessile ridges in most terebelliforms and the outgroup (-0), with the hooks or uncini being embedded in a furrow (e.g. Figure A3B,F). However, in Pectinariidae and species of *Amphicteis* the anterior tori form distinct lobes (-1, Figure A2D) that are fin-like and emerge from the body wall. The uncini are arranged along the distal margin of these lobes.

72. Posterior neuropodia tori, elevation from body wall.

In most of the ingroup, the tori become more developed on the abdomen and form distinct fin-like lobes (-1, Figure A2a). However, in the Polycirrini *Amaeana* and *Polycirrus*, the Terebellini *Terebella lapidaria* and *Spinosphaera hutchingsae*, and the Alvinellidae the uncinigers are low throughout the body (-0, Figure A1a).

73. Type of neurochaetae in anterior segments.

In the outgroup, long-handled hooks are present throughout the entire body. Hooks are present in the thorax of Trichobranchidae (-0). Most Terebelliformia have short-handled uncini throughout (-1). Several groups have two different types of neurochaetae in their anterior body; Melinnidae have up to four segments with aciculae before the start of uncini (-2); *Trichobranchus* has geniculate aciculae followed by the characteristic trichobranchid hooks (-3).

74. Type of neurochaetae in posterior segments.

The posterior part of the body carries uncini in most species (-0). In Pectinariidae, the abdomen (scaphe) is achaetous except for the first segment that carries aciculae (-1). *Amaeana trilobata* (Polycirrini) differs from all other terebellids in not having uncini but acicular neuropodia throughout the posterior part of its body (-1).

75. Organization of capitium in uncini or hooks of anterior segments.

The outgroup, Trichobranchidae and Terebellidae are characterized by hooks or uncini with the small teeth of the capitium arched in multiple rows cresting the rostrum (-0). Ampharetidae, Melinnidae and Pectinariidae have their teeth on uncini arranged in separate vertical columns rather than rows. Their uncini are pectinate, i.e. having columns of similar sized teeth above each other in one (-2), two (-3), three (-4), or more than three (-5) parallel columns. Adult Alvinellidae have a characteristic type of uncinus with a single prominent tooth over the rostrum (-1, Figure A1E).

76. Organization of capitium in uncini of posterior segments.

Where uncini are present in the posterior body, the same states as in character 75 apply.

77. Rostrum of the uncini or hooks.

The rostrum is the prominent tooth below the capitium present in the hooks of the outgroup and the uncini and hooks of Terebellidae and Trichobranchidae (-0). The rostrum is formed earlier than the secondary teeth of the capitium and is therefore an independent structure [61]. Pectinariids were shown to not have a rostrum (-1; [62]) and the same is assumed for ampharetids and melinnids, even though ultrastructural confirmation is still lacking. The large tooth present in uncini of Alvinellidae could be a rostrum (Figure A1e). On the other hand, it could be a greatly enlarged secondary tooth similar to the situation in ampharetids. Until their development is studied, we treat alvinellid uncini as possessing a rostrum (-0).

78. Size of the subrostrum in uncini or hooks of anterior segments.

The subrostrum is the area below the rostrum. It can be separated into an upper and lower subrostrum if a subrostral process is present (cf. character 77). The hooks of the outgroup and Trichobranchidae have a small subrostrum (-0) atop the greatly elongate basis. Even though Ampharetinae, Melinnidae and Pectinariidae lack a rostrum and therefore strictly lack a 'subrostrum', we still consider the area below the lowest tooth as a subrostrum. The uncini of Ampharetidae, Melinnidae, Pectinariidae and a several Terebellidae (e.g. *Proclea malmgreni*) have shorter subrostra that are either about the same length as the capitium or shorter (-0). A long subrostrum is present in Alvinellidae and many

Terebellidae (-1). The subrostrum and the anterior process can form a long projection that extends further than the capitium and rostrum (-1) as in Alvinellidae (Figure A1E) and all Thelepodinae, Polycirrini and some other Terebellinae.

79. Size of the subrostrum in uncini of posterior segments.

Pectinariidae were scored with non-applicable (-) as they have aciculae on posterior segments, not uncini. For the terminals with uncini on posterior segments, the same states as for character 78 apply.

80. Subrostral process in uncini or hooks of anterior segments.

The subrostral process is a knob-like process on the subrostrum. In the outgroup and all Trichobranchidae sampled here, a subrostral process is not present on hooks (-0). The subrostral process can be prominent (-1), as in Pectinariidae, where it can be spiny. Note that [3] view the subrostral spines of Pectinariidae as parts of the capitium and therefore consider the subrostral process as absent in this group. We do not agree with this interpretation as the subrostral spines are below the capitium and should therefore be considered as separate. The subrostral process is also prominent in Thelepodinae, where it forms a rounded knob that can even emerge from the body wall (see fig. 39c in [3]). All Melinnidae have a subrostral process. It is absent in Alvinellidae (-0, Figure A1d) and most Ampharetidae.

81. Subrostral process in uncini of posterior segments.

For terminals with uncini on posterior segments the same options as for character 80 apply.

82. Position of subrostral process in uncini or hooks of anterior segments (subordinate to character 80-1).

If present, the subrostral process is located either near the capitium or rostrum (-0), medially on the subrostrum (-1), or near the anterior process (-2). In some Pectinariidae, the subrostrum is small so it is entirely spanned by the spiny subrostral appendix (-3).

83. Position of subrostral process in uncini of posterior segments (subordinate to character 81-1).

For terminals with uncini on posterior segments, the same options as for character 80 apply except for state -3 as Pectinariidae lack uncini on posterior segments.

84. Shape of the anterior process in uncini of anterior segments.

The anterior process is only present in uncini, not in hooks. It serves as the attachment for a ligament and it can be pointed (-0, Figure A1E) or rounded (-1).

85. Shape of the anterior process in uncini of posterior segments.

For terminals with uncini on posterior segments, the same states as for character 84 apply.

86. Beard surrounding the teeth of the hooks or uncini.

In larval and recently settled *Arenicola marina*, several hair-like structures originate from the shaft and surround the tip of the rostrum (-0). The beard appears in hooks of juveniles of the arenicolid *Arenicola marina* [61,63]. It is absent (-1) in all Terebelliformia.

Terminal part of the body.

Pygidium.

87. Pygidial cirri.

The outgroup and most terebelliforms have a smooth or papillate anal rim (-0). However, long anal cirri are present in several Ampharetidae (e.g. *Samytha sexcirrate*, Figure 2b), members of the Pectinariidae, and *Octobranchus* (Trichobranchidae) (-1).

88. Pygidial eyespots.

Eyespots on the pygidium are absent among most taxa (-0), but present in *Mugga wahrbergi, Melinna oculata* and *Petta pusilla* (-1).

89. Life style.

The outgroup, Polycirrini and *Trichobranchus* species are free-living and crawl over the sediment or are burrowing (-0). Most terebelliforms build tubes using their lips and tentacles and live permanently in those (-1). *Biremis* cf. *blandi* is free living and capable of swimming to avoid predation by wiggling its body [34]. The proboscis-bearing members of *Trichobranchus* use their proboscii to burrow through the sediment. In most cases, a burrowing life style is associated with the absence of a tube, except for the terebellid *Artacama* that can dig with its proboscis but also builds tubes on the sediment surface ({Holthe:1986tn} the other one! /fig. 38d). Information on the life-history of the other Trichobranchidae is sparse and has therefore been coded as unknown, only *Terebellides stroemii* has been reported to live in thin mucous tubes encrusted with mud [64]. Pectinariidae dig head-down through the sediment using their paleae while in their tubes [30].

90. Consistency of the tube.

The tubes are mostly soft (-0), mostly made of mud, sand, and shell fragments (Figure 2c, d). Pectinariidae build beautiful rigid tubes from sand grains and shell fragments (-1).

References

1. Rouse, G.; Pleijel, F. *Polychaetes*; OUP Oxford, 2001; ISBN 9780198506089.
2. Zhadan, A.E.; Tzetlin, A.B. Comparative morphology of the feeding apparatus in the Terebellida (Annelida: Polychaeta). *Cah. Biol. Mar.* **2002**.
3. Nogueira, J.M.D.M.; Hutchings, P.A.; Fukuda, M.V.; Others Morphology of terebelliform polychaetes (Annelida: Polychaeta: Terebelliformia), with a focus on Terebellidae. *Zootaxa* **2010**, 1–185.
4. de Matos Nogueira, J.M.; Fitzhugh, K.; Hutchings, P. The continuing challenge of phylogenetic relationships in Terebelliformia (Annelida : Polychaeta). *Invertebr. Syst.* **2013**, *27*, 186–238.
5. Wells, G.P. The proboscis apparatus of *Arenicola*. *J. Mar. Biol. Assoc. U. K.* **1952**, *31*, 1–28.
6. Hartman, O. Endemism in the North Pacific Ocean, with emphasis on the distribution of marine annelids, and descriptions of new or little known species. *Essays in the natural sciences in honor of Captain Allan Hancock* **1955**, 39–60.
7. Holthe, T. The systematic position of *Artacamella* Hartman, 1955 (Polychaeta, Terebellomorpha). *Sarsia* **1977**, *63*, 35–37.
8. Garraffoni, A.R.S.; da Cunha Lana, P. Cladistic analysis of the subfamily Trichobranchinae (Polychaeta: Terebellidae). *J. Mar. Biol. Assoc. U. K.* **2004**, *84*, 973–982.
9. Garraffoni, A.R.S.; Lana, P.C. Is *Filibranchus*, Malm 1874 (Trichobranchidae: Polychaeta) A Natural Taxon? *Sarsia* **2002**, *87*, 472–477.
10. Muir, A.I. A redescription of *Unobranchus* (Annelida: Terebellida: Trichobranchidae), with notes on the systematics of Trichobranchidae. *Ital. J. Zool.* **2011**, *78*, 140–147.
11. Zhadan, A.E.; Tzetlin, A.B. Comparative study of the diaphragm (gular membrane) in Terebelliformia (Polychaeta, Annelida). In Proceedings of the Advances in Polychaete Research; Springer Netherlands, 2003; pp. 269–278.
12. Wirén, A. *Om Cirkulations-och Digestions-Organen: Hos Annelider af Familjerna Ampharetidæ, Terebellidæ och Amphictenidæ*; Kongl. Boktryckeriet, 1885; Vol. 21.
13. Garraffoni, A.R.S.; Lana, P.C. Phylogenetic relationships within the Terebellidae (Polychaeta : Terebellida) based on morphological characters. *Invertebr. Syst.* **2009**, *22*, 605–626.
14. Garraffoni, A.R.S.; Lana, P.C. A critical review of ontogenetic development in Terebellidae (Polychaeta). *Acta Zool.* **2010**, *91*, 390–401.
15. Holthe, T. Polychaeta Terebellomorpha. Marine Invertebrates of Scandinavia. Vol. 7. *Norwegian University: Trondheim, Norway* **1986**.
16. Storch, V.; Gaill, F. Ultrastructural observations on feeding appendages and gills of *Alvinella pompejana* (Annelida, Polychaeta). *Helgoländer Meeresuntersuchungen* **1986**, *40*, 309.
17. Welsh, J.H. The Structure and Reactions of the Tentacles of *Terebella*

magnifica W. *Biol. Bull.* **1934**, *66*, 339–345.
18. Zal, F.; Desbruyeres, D.; Jouin-Toulmond, C. Sexual dimorphism in *Paralvinella grasslei*, a polychaete annelid from deep-sea hydrothermal vents. *Comptes rendus de l'Académie des sciences. Série 3, Sciences de la vie* **1994**, *317*, 42–48.
19. Pradillon, F.; Gaill, F. Oogenesis characteristics in the hydrothermal vent polychaete *Alvinella pompejana*. *Invertebr. Reprod. Dev.* **2003**, *43*, 223–235.
20. Zhadan, A.E.; Tsetlin, A.B.; Safronova, M.A. Anatomy of some representatives from the family Alvinellidae (Polychaeta, Terebellida) from the Pacific hydrothermal habitats. *Зоологический журнал* **2000**, *79*, 159–160.
21. Jouin-Toulmond, C.; Zal, F.; Hourdez, S. Genital apparatus and ultrastructure of the spermatozoa in *Alvinella pompejana* (Annelida: Polychaeta). *Cah. Biol. Mar.* **1997**.
22. Garraffoni, A.R.; Lana, P.C. Análise cladística do gênero *Octobranchus* Marion & Bobretzky, 1875 (Trichobranchidae: Polychaeta). *Braz. J. Aquat. Sci. Technol.* **2010**, *4*, 43–48.
23. Holthe, T. *Evolution, systematics, and distribution of the Polychaeta Terebellomorpha, with a catalogue of the taxa and a bibliography*; NTNU Vitenskapsmuseet, 1986;.
24. Reuscher, M.; Fiege, D.; Wehe, T. Terebellomorph polychaetes from hydrothermal vents and cold seeps with the description of two new species of Terebellidae (Annelida: Polychaeta) representing the first records of the family from deep-sea vents. *J. Mar. Biol. Assoc. U. K.* **2012**, *92*, 997–1012.
25. Nogueira, J.M. de M.; Carrerette, O.; Hutchings, P.; Fitzhugh, K. Systematic review of the species of the family Telothelepodidae Nogueira, Fitzhugh & Hutchings, 2013 (Annelida, Terebelliformia), with descriptions of three new species. *Mar. Biol. Res.* **2018**, *14*, 217–257.
26. Hilbig, B. Family Ampharetidae Malmgren, 1867. *Taxonomic atlas of the benthic fauna of the Santa Maria Basin and Western Santa Barbara Channel. The Annelida Part* **2000**, *4*, 169–230.
27. Jouin-Toulmond, C.; Hourdez, S. Morphology, ultrastructure and functional anatomy of the branchial organ of *Terebellides stroemii* (Polychaeta: Trichobranchidae) and remarks on the systematic position of the genus *Terebellides*. *Cah. Biol. Mar.* **2006**, *47*, 287–299.
28. Kingston, P.F.; Mackie, A.S.Y. *Octobranchus floriceps* sp. nov. (Polychaeta: Trichobranchidae) from the northern North Sea with a reexamination of *O. antarcticus* Monro. *Sarsia* **1980**.
29. Hutchings, P.; Peart, R. A revision of the Australian Trichobranchidae (Polychaeta). *Invertebr. Syst.* **2000**, *14*, 225–272.
30. Watson, A.T. Observations on the habits and life-history of *Pectinaria* (*Lagis*) *koreni*, Mgr. *Proc. Trans. Lpool biol. Soc.* **1928**, *42*, 25–60.
31. Truchet, M.; Vovelle, J. Etude de la glande cémentaire d'un Polychète tubicole (*Pectinaria* (=*Lagis*) *koreni*) à l'aide de la microsonde électronique, du microanalyseur ionique et du microscope électronique à balayage. *Calcif.*

Tissue Res. **1977**, *24*, 231–238.
32. Hessle, C. *Zur kenntnis der terebellomorphen polychaeten.*; Almqvist & Wiksells boktryckeri-a.-b., 1917; Vol. 5.
33. Londono-Mesa, M.H. Terebellidae (Polychaeta: Terebellida) from the Grand Caribbean region. *Zootaxa* **2009**, *2320*, 1–93.
34. Polloni, P.T.; Rowe, G.T.; Teal, J.M. *Biremis blandi* (Polychaeta: Terebellidae), new genus, new species, caught by DSRV "Alvin" in the Tongue of the Ocean, New Providence, Bahamas. *Mar. Biol.* **1973**, *20*, 170–175.
35. Schüller, M.; Hutchings, P.A. New species of *Terebellides* (Polychaeta: Trichobranchidae) indicate long-distance dispersal between western South Atlantic deep-sea basins. *Zootaxa* **2012**, *3254*, 1–31.
36. Schüller, M.; Hutchings, P.A. New species of *Terebellides* (Polychaeta: Trichobranchidae) from the deep Southern Ocean, with a key to all described species. *Zootaxa* **2013**, *3619*, 1–45.
37. Gamble, F.W.; Ashworth, J.H. The habits and structure of *Arenicola marina. Stud. Biol. Biol. Dep. Owens College* **1899**, *4*, 87.
38. British Museum (Natural History).; Day, J.H. *A monograph on the Polychaeta of southern Africa.* London: **1967**.
39. Jouin-Toulmond, C.; Mozzo, M.; Hourdez, S. Ultrastructure of spermatozoa in four species of Alvinellidae (Annelida: Polychaeta). *Cah. Biol. Mar.* **2002**, *43*, 391–394.
40. Salazar-Vallejo, S.I.; Hutchings, P. A review of characters useful in delineating ampharetid genera (Polychaeta). *Zootaxa* **2012**, *3402*, 45–53.
41. Reuscher, M.; Fiege, D.; Wehe, T. Four new species of Ampharetidae (Annelida: Polychaeta) from Pacific hot vents and cold seeps, with a key and synoptic table of characters for all genera. *Zootaxa* **2009**.
42. Hutchings, P.; Peart, R. A review of the genera of Pectinariidae (Polychaeta) together with a description of the Australian fauna. *Rec. Aust. Mus.* **2002**, *54*, 99–127.
43. Hutchings, P.; Glasby, C.J. The Amphitritinae (Polychaeta: Terebellidae) from Australia. *Rec. Aust. Mus.* **1988**, *40*, 1–60.
44. Fauchald, K. The polychaete worms. Definitions and keys to the orders, families and genera. *Natural History Museum of Los Angeles Country, Science Series* **1977**, *28*, 1–190.
45. Day, J.H. A review of the family Ampharetidae (Polychaeta). *Ann. S. Afr. Mus.* **1964**, *48*, 97–120.
46. Wells, G.P. The parapodia of *Arenicola marina* L.(Polychaeta). *Proc. Zool. Soc. Lond.* **1944**, *114*, 100–116.
47. Fauvel, P. *Faune de France: Polychètes sédentaires*; Paul Lechevalier, 1927.
48. Long, C.D. Pectinariidae (Polychaeta) from Caribbean and Associated Waters. *Bull. Mar. Sci.* **1973**, *23*, 857–874.
49. Ashworth, J.H. Memoir on *Arenicola*. The lugworm. *Proc. Trans. Lpool biol. Soc.* **1904**, *18*, 209–326.
50. Jirkov, I.A. Revision of Ampharetidae (Polychaeta) with modified thoracic notopodia. *Invertebrate Zoology* **2008**, *5*, 111–132.

51. Grelon, D.; Morineaux, M.; Desrosiers, G.; Juniper, S.K. Feeding and territorial behavior of *Paralvinella sulfincola*, a polychaete worm at deep-sea hydrothermal vents of the Northeast Pacific Ocean. *J. Exp. Mar. Bio. Ecol.* **2006**, *329*, 174–186.
52. Rousset, V.; Rouse, G.W.; Feral, J.-P.; Desbruyeres, D.; Pleijel, F. Molecular and morphological evidence of Alvinellidae relationships (Terebelliformia, Polychaeta, Annelida). *Zool. Scr.* **2003**, *32*, 185–197.
53. Jirkov, I.A. Discussion of taxonomic characters and classification of Ampharetidae (Polychaeta). *Ital. J. Zool.* **2011**, *78*, 78–94.
54. Orrhage, L. On the anatomy of the central nervous system and the morphological value of the anterior end appendages of Ampharetidae, Pectinariidae and Terebellidae (Polychaeta). *Acta Zool.* **2001**, *82*, 57–71.
55. Imajima, M.; Reuscher, M.G.; Fiege, D. Ampharetidae (Annelida: Polychaeta) from Japan. Part II: Genera with elevated and modified notopodia. *Zootaxa* **2013**, *3647*, 137–166.
56. Bhaud, M.; Gremare, A. Larval development of the terebellid polychaete *Eupolymnia nebulosa* (Montagu) in the Mediterranean Sea. *Zool. Scr.* **1988**, *17*, 347–356.
57. Blake, J.A. Larval Development of Polychaeta from the Northern California Coast V. *Ramex californiensis* Hartman (Polychaeta: Terebellidae). *Bull. Mar. Sci.* **1991**, *48*, 448–460.
58. Garraffoni, A.R.S.; Amaral, A.C.Z. Postlarval development of Nicolea uspiana (Polychaeta: Terebellidae). *Zoologia* **2009**, *26*, 61–66.
59. Capa, M.; Hutchings, P.A. Terebellidae (Polychaeta) from Coiba National Park, Panamanian Pacific, including description of four new species and synonymy of the genus *Paraeupolymnia* with *Lanicola*. *Zootaxa* **2006**, *1375*, 1–29.
60. Hartman, O. *Reteterebella queenslandia*, a new genus and species of polychaetous annelid from Queensland, Australia. *Rec. Aust. Mus.* **1963**, *25*, 355–357.
61. Hausen, H. Chaetae and chaetogenesis in polychaetes (Annelida). In *Morphology, Molecules, Evolution and Phylogeny in Polychaeta and Related Taxa*; Bartolomaeus, T., Purschke, G., Eds.; Springer Netherlands: Dordrecht, 2005; pp. 37–52 ISBN 9781402032400.
62. Bartolomaeus, T. Structure and formation of the uncini in *Pectinaria koreni*, *Pectinaria auricoma* (Terebellida) and *Spirorbis spirorbis* (Sabellida): inplications for annelid phylogeny and the position of the Pogonophora. *Zoomorphology* **1995**, *115*, 161–177.
63. Bartolomaeus, T.; Meyer, K. Development and Phylogenetic Significance of Hooked Setae in Arenicolidae (Polychaeta, Annelida). *Invertebr. Biol.* **1997**, *116*, 227–242.
64. Michel, C.; Bhaud, M.; Boumati, P.; Halpern, S. Physiology of the digestive tract of the sedentary polychaete *Terebellides stroemi*. *Mar. Biol.* **1984**, *83*, 17–31.
